# Morbidity and Mortality of Patients Who Underwent Minimally Invasive Esophagectomy After Neoadjuvant Chemoradiotherapy vs Neoadjuvant Chemotherapy for Locally Advanced Esophageal Squamous Cell Carcinoma: A Randomized Clinical Trial

**DOI:** 10.1001/jamasurg.2021.0133

**Published:** 2021-03-17

**Authors:** Hao Wang, Han Tang, Yong Fang, Lijie Tan, Jun Yin, Yaxing Shen, Zhaochong Zeng, Jiangyi Zhu, Yingyong Hou, Ming Du, Jia Jiao, Hongjing Jiang, Lei Gong, Zhigang Li, Jun Liu, Deyao Xie, Wenfeng Li, Changhong Lian, Qiang Zhao, Chun Chen, Bin Zheng, Yongde Liao, Kuo Li, Hecheng Li, Han Wu, Liang Dai, Ke-Neng Chen

**Affiliations:** 1 Department of Thoracic Surgery, Zhongshan Hospital, Fudan University, Shanghai, China; 2 Department of Radiotherapy, Zhongshan Hospital, Fudan University, Shanghai, China; 3 Department of Pathology, Zhongshan Hospital, Fudan University, Shanghai, China; 4 Department of Cardiothoracic Surgery, The First Affiliated Hospital of Chongqing Medical University, Chongqing, China; 5 Department of Esophageal Cancer, Tianjin Medical University Cancer Institute and Hospital, Tianjin, China; 6 Department of Thoracic Surgery, Shanghai Chest Hospital, Shanghai Jiao Tong University, Shanghai, China; 7 Department of Radiotherapy, Shanghai Chest Hospital, Shanghai Jiao Tong University, Shanghai, China; 8 Department of Thoracic Surgery, The First Affiliated Hospital of Wenzhou Medical University, Wenzhou, Zhejiang, China; 9 Department of Radiation Oncology, The First Affiliated Hospital of Wenzhou Medical University, Wenzhou, Zhejiang, China; 10 Department of General Surgery, Heping Hospital Affiliated to Changzhi Medical College, Changzhi, Shanxi, China; 11 Department of Thoracic Surgery, Fujian Medical University Union Hospital, Fuzhou, Fujian, China; 12 Department of Thoracic Surgery, Union Hospital, Tongji Medical College, Huazhong University of Science and Technology, Wuhan, Hubei, China; 13 Department of Thoracic Surgery, Ruijin Hospital, Shanghai Jiao Tong University School of Medicine, Shanghai, China; 14 Key Laboratory of Carcinogenesis and Translational Research (Ministry of Education), The First Department of Thoracic Surgery, Peking University Cancer Hospital and Institute, Peking University School of Oncology, Beijing, China

## Abstract

**Importance:**

Safety and efficacy of neoadjuvant chemoradiotherapy (nCRT) vs neoadjuvant chemotherapy (nCT) for treatment of locally advanced esophageal squamous cell carcinoma (ESCC) remain uncertain given lack of high-level clinical evidence.

**Objective:**

To compare safety and long-term survival of nCRT followed by minimally invasive esophagectomy (MIE) with that of nCT followed by MIE for patients with locally advanced ESCC.

**Design, Setting, and Participants:**

A prospective, multicenter, open-label, randomized clinical trial that compared safety and efficacy of nCRT vs nCT followed by MIE for patients with locally advanced ESCC. From January 1, 2017, to December 31, 2018, 264 patients with ESCC of clinical stages from cT3 to T4aN0 to 1M0 were enrolled. Analysis was performed on an intention-to-treat basis from January 1, 2017, to August 30, 2020.

**Interventions:**

Eligible patients were randomized to the nCRT group (n = 132) or the nCT group (n = 132) by a computer-generated random system. The chemotherapy, based on paclitaxel and cisplatin, was administered to both groups, while 40 Gy of concurrent radiotherapy was added for the nCRT group. At about 6 weeks after neoadjuvant therapy, MIE via thoracoscopy and laparoscopy was performed for the patients in both groups.

**Main Outcomes and Measures:**

The primary outcome was 3-year overall survival. Secondary outcomes included postoperative complications, mortality, postoperative pathologic outcome, recurrence-free survival time, and quality of life.

**Results:**

Among 264 patients (226 men [85.6%]; mean [SD] age, 61.4 [6.8] years), postoperative morbidity was 47.4% in the nCRT group (54 of 114) and 42.6% in the nCT group (46 of 108), with no significant difference between groups (difference, 4.8%; 95% CI, −8.2% to 17.5%; *P* = .48). Distribution of the severity of complications was similar between the 2 groups based on Clavien-Dindo classification. The 90-day perioperative mortality rate was 3.5% for the nCRT group (4 of 114) and 2.8% for the nCT group (3 of 108) (*P* = .94). The R0 resection rates were similar between groups (109 of 112 [97.3%] vs 100 of 104 [96.2%]; *P* = .92). However, patients in the nCRT group had a higher pathologic complete response (residual tumor, 0%) rate (40 of 112 [35.7%] vs 4 of 104 [3.8%]; *P* < .001) and a higher rate of negative lymph nodes (ypN0, 74 of 112 [66.1%] vs 48 of 104 [46.2%]; *P* = .03) than those in the nCT group. One-year overall survival using intention-to-treat analysis was 87.1% in the nCRT group (115 of 132) and 82.6% in the nCT group (109 of 132) (*P* = .30). Furthermore, deaths caused by tumor progression or recurrence were significantly less in the nCRT group than in the nCT group (9 of 132 [6.8%] vs 19 of 132 [14.4%]; *P* = .046); however, deaths from nontumor causes were similar (8 of 132 [6.1%] vs 4 of 132 [3.0%]; *P* = .24).

**Conclusions and Relevance:**

Initial results of the trial showed that nCRT followed by MIE has similar safety to and better histopathologic outcome than nCT followed by MIE for treatment of locally advanced ESCC.

**Trial Registration:**

ClinicalTrials.gov Identifier: NCT03001596

## Key Points

**Question:**

Is there any difference in the safety of neoadjuvant chemoradiotherapy (nCRT) followed by minimally invasive esophagectomy (MIE) for locally advanced esophageal squamous cell carcinoma (ESCC) compared with that of neoadjuvant chemotherapy (nCT) followed by MIE?

**Findings:**

In this multicenter randomized clinical trial of 264 patients with ESCC, overall morbidity rates were 47% in the nCRT group and 43% in nCT group, which was not significantly different.

**Meaning:**

This trial shows that the safety of nCRT followed by MIE is similar to that of nCT for the treatment of locally advanced ESCC.

## Teaser

This randomized clinical trial compares the safety and long-term survival of neoadjuvant chemoradiotherapy followed by minimally invasive esophagectomy (MIE) with that of neoadjuvant chemotherapy followed by minimally invasive esophagectomy for patients with locally advanced esophageal squamous cell carcinoma (ESCC).

## Introduction

Esophagectomy remains the cornerstone of current therapy for esophageal cancer, one of the most common cancers worldwide. However, the surgery alone is usually accompanied by high recurrence or metastasis rates with poor survival among patients with locally advanced esophageal cancer.^1^ Therefore, multidisciplinary therapy has been strongly recommended to improve the prognosis.

Currently, more and more evidence has suggested the survival benefit from neoadjuvant therapy followed by surgery for locally advanced esophageal cancer. The effectiveness of neoadjuvant chemoradiotherapy (nCRT) followed by surgery has been well established by CROSS (Chemoradiotherapy for Oesophageal Cancer Followed by Surgery Study) and other trials.^2,3,4^ Meanwhile, the effectiveness of neoadjuvant chemotherapy (nCT) followed by surgery has also been demonstrated in several other trials.^5,6,7^ Nevertheless, there are only 3 clinical trials available directly comparing nCRT with nCT for esophageal cancer so far, to our knowledge.^8,9,10^ Moreover, the cases in these studies were all or predominantly cases of adenocarcinoma located in the distal esophagus or esophagogastric junction. Thus, whether the results could be extrapolated for patients with esophageal squamous cell carcinoma (ESCC) remains to be validated.

On the other hand, a meta-analysis reported that nCRT was significantly associated with increased risk of perioperative morbidity or mortality for patients with ESCC, which may impose restrictions on the application of nCRT.^11^ However, the esophagectomies performed in these trials were open surgical procedures; the amount of trauma caused by open surgery contributes to the high rates of morbidity and mortality. Minimally invasive esophagectomy (MIE) has the advantage of less trauma, quicker recovery, improved quality of life, and equal oncologic survival.^12,13,14^ However, this advanced technique has not been widely applied for patients after neoadjuvant therapy owing to high demand in surgical skills. Thus, whether MIE could be beneficial after neoadjuvant therapy remains to be clarified.

A retrospective pilot study discovered that patients with locally advanced ESCC who underwent nCRT followed by MIE had similar mortality and morbidity as patients who underwent nCT, as well as better 3-year overall survival.^15^ To further critically evaluate the safety and long-term oncologic survival of patients who undergo nCRT vs nCT followed by MIE for locally advanced ESCC, we launched this prospective, multicenter, randomized clinical trial in January 2017. Accordingly, we present the morbidity and mortality results of this trial.

## Methods

### Study Design

This study was a prospective, multicenter, parallel, open-label, randomized clinical trial conducted from January 1, 2017, to December 31, 2018. Ten high-volume institutions in China participated in the study. The primary outcome was 3-year overall survival. The secondary end points included postoperative complications, mortality, postoperative pathologic outcome, recurrence-free survival time, and quality of life. The trial protocol is in Supplement 1.^16^ Approval was obtained from the ethics committee of the Zhongshan Hospital and from the institutional review board at each institution (Zhongshan Hospital, Fudan University, Shanghai, China; The First Affiliated Hospital of Chongqing Medical University, Chongqing, China; Tianjin Medical University Cancer Institute and Hospital, Tianjin, China; Shanghai Chest Hospital, Shanghai Jiao Tong University, Shanghai, China; The First Affiliated Hospital of Wenzhou Medical University, Wenzhou, China; Heping Hospital Affiliated to Changzhi Medical College, Changzhi, Shanxi, China; Fujian Medical University Union Hospital, Fuzhou, Fujian, China; Union Hospital, Tongji Medical College, Huazhong University of Science and Technology, Wuhan, Hubei, China; Ruijin Hospital, Shanghai Jiao Tong University School of Medicine, Shanghai, China; and Peking University Cancer Hospital and Institute, Peking University School of Oncology, Beijing, China). All included patients provided written informed consent. This trial has been registered and released in ClinicalTrials.gov (identifier NCT03001596). This study followed the Consolidated Standards of Reporting Trials (CONSORT) reporting guideline.

### Eligibility

Patients with histologically confirmed, potentially curable squamous cell carcinoma were eligible for inclusion in the study. The upper border of the tumor had to be at least 3 cm below the upper esophageal sphincter. Imaging examinations, including thoracoabdominal enhanced computed tomography, cervical ultrasonography, endoscopic ultrasonography (performed when possible), and positron emission tomography (optional when necessary), were used to determine the clinical stage. Only patients with tumors of clinical stages from T3 to T4aN0 to N1 and no clinical evidence of metastatic spread (M0), according to the International Union Against Cancer Tumor, Node, Metastasis (TNM) Classification (8th edition),^17^ were enrolled. Eligible patients were 18 to 75 years of age, had an Eastern Cooperative Oncology Group performance status score of 2 or lower (range, 0-5, with 0 indicating fully active, 1 indicating unable to carry out heavy physical work, and 2 indicating up and about more than half the day but unable to work), and had lost 10% or less of body weight. Patients also had to have adequate hematologic, kidney, liver, and pulmonary function, as well as no history of other cancer or radiotherapy or chemotherapy. The details of eligibility criteria are in eTable 1 in Supplement 2.

### Randomization

Patients were randomly assigned in a 1:1 allocation ratio to receive nCRT followed by MIE (nCRT group) or nCT followed by MIE (nCT group) and were stratified according to coordinating centers. Randomization was assigned by the computer-generated random system in the Biomedical Statistics Center, Fudan University. Each assignment was generated after the completion of patient registration in the random system online.

### Pretreatment Workup and Staging

All patients underwent pretreatment staging. This included obtaining history; physical examination; pulmonary function tests; routine hematologic and biochemical tests; upper gastrointestinal endoscopy with histologic biopsy and endoscopic ultrasonography; contrast-enhanced computed tomography of the neck, chest, and upper abdomen; and external ultrasonography of the neck, with fine-needle aspiration of lymph nodes when cancer was suspected. For the final analysis, the available endoscopic reports were reviewed. Positron emission tomography and radionuclide bone imaging were also performed when necessary.

### Treatment

#### Neoadjuvant Chemoradiotherapy

On days 1, 8, 15, and 22, paclitaxel, 50 mg/m^2^, and cisplatin, 25 mg/m^2^ of body surface area, were administered intravenously. A total radiotherapy dose of 40 Gy was administered in 20 fractions of 2 Gy, 5 fractions per week, starting the first day of chemotherapy. All patients were treated with external beam radiotherapy.

#### Neoadjuvant Chemotherapy

The nCT group consists of 2 cycles of preoperative chemotherapy. The regimen was intravenous paclitaxel, 135 mg/m^2^, and cisplatin, 75 mg/m^2^, on day 1. The second cycle was given after 3 weeks.

### Assessments During Neoadjuvant Treatment

Patients were closely monitored for toxic effects of chemotherapy with the use of the National Cancer Institute’s Common Terminology Criteria for Adverse Events, version 5.0.^18^ Vital signs, body weight, description of symptoms, and results of standard laboratory tests (complete blood count and blood biochemistry) were obtained and recorded weekly before and during the neoadjuvant therapy period to assess the toxic effects of preoperative therapy. After 4 weeks of neoadjuvant therapy, computed tomography (or positron emission tomography–computed tomography) of the thorax and abdomen and ultrasonography of the neck were performed to restage the tumor.

### Surgical Procedure

At about 6 weeks after neoadjuvant therapy, MIE via thoracoscopy and laparoscopy was performed for the patients in both groups.^19^ To achieve an accurate ypTNM stage, an extensive mediastinal lymph node dissection, including a bilateral laryngeal recurrent nerve lymph node dissection, was requested for every patient.^20^ Dissected abdominal nodes included the paracardia, lesser curvature, greater curvature, left gastric, common hepatic, splenic, and celiac lymph nodes. For tumors located at the upper one-third of the esophagus, a bilateral cervical lymph node dissection was added to reach a 3-field lymph node dissection. Gastric tube reconstruction with a cervical anastomosis was the preferred technique for restoring continuity of the digestive tract.

### Outcome Measurements for Surgical Safety Analysis

Morbidity and mortality were examined within 90 days after surgery. The definition of complication was based on the International Consensus on Standardization of Data Collection for Complications Associated With Esophagectomy.^21^ A specific complication was diagnosed on the basis of either obvious clinical evidence or an image-based physical evaluation. The severity of postoperative complications was assessed according to the Clavien-Dindo classification of surgical complications. Analysis of the causes of death in both groups in the first year after randomization was performed to clarify whether death was related to complications from the modality of treatment or tumor recurrence.

### Assessments During Follow-up

The first follow-up visit was 1 month after surgery. From then on, follow-up visits were every 3 months in the first 2 years after surgery and every 6 months from the third year until the end of the trial or death. The end of the trial will be at least 3 years after the treatment of the last patient. The detailed examination items included standard laboratory tests (complete blood count and tumor biomarkers), computed tomography of the thorax and abdomen, ultrasonography of the neck, and esophagogastroduodenoscopy whenever indicated.

### Sample Size

The sample size calculations were based on the primary outcome of overall survival. The 3-year overall survival rate in a previous report was about 77% in the nCRT group and 50% in the nCT group, without differences in mortality.^15^ Therefore, the total sample size was calculated to be 264, which was based on the intention of showing a benefit of nCRT vs nCT in the primary end point of 20% with a 1-sided type I error of 5% and a power of 90%, as well as a 15% dropout rate before surgery or loss to follow-up according to power analysis and sample size. Thus, 132 patients were enrolled in each group according to 1:1 randomized allocation.

### Statistical Analysis

Data analysis was performed according to the intention-to-treat principle for all randomized patients from January 1, 2017, to August 30, 2020. Statistical analysis was undertaken using SPSS, version 23.0 (IBM Corp). Comparisons between the 2 groups were performed using the χ^2^ test and the Fisher exact test for categorical parameters, and the *t* test or analysis of variance was used for continuous variables. A 2-sided *P* < .05 was considered to be statistically significant.

## Results

### Patients

Between January 2017 and December 2018, a total of 416 patients in 10 high-volume centers in China were assessed, and 264 patients (226 men [85.6%]; mean [SD] age, 61.4 [6.8] years) were enrolled and randomly allocated to the nCRT group (n = 132) or the nCT group (n = 132) (Figure). The baseline clinical characteristics of the patients enrolled were well balanced (eTable 2 in Supplement 2). There were 78 cases (37 in nCRT group and 41 in nCT group) of cT4a disease in this trial. Structures invaded were the pleura (29 in nCRT group and 32 in nCT group), pericardium (3 in nCRT group and 2 in nCT group), and diaphragm crus (5 in nCRT group and 7 in nCT group).

**Figure.**
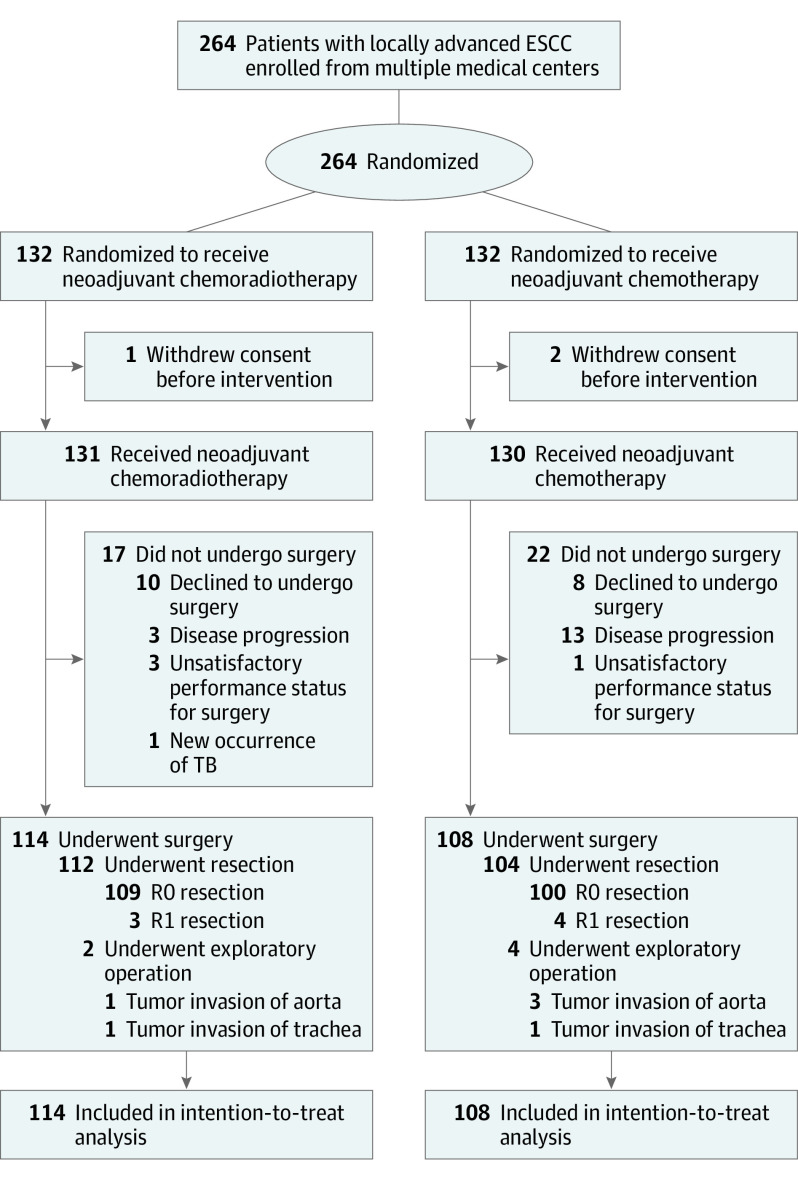
CONSORT Diagram ESCC indicates esophageal squamous cell carcinoma; TB, tuberculosis.

### Neoadjuvant Treatment

Three patients withdrew consent before treatment (1 in nCRT group and 2 in nCT group). Thus, 131 patients in nCRT group and 130 patients in nCT group received neoadjuvant treatment. Adverse events during neoadjuvant treatment are shown in eTable 3 in Supplement 2. The occurrence of grade 3 and grade 4 adverse events was higher in the nCRT group than in nCT group (20 of 131 [15.3%] vs 9 of 130 [6.9%]; *P* = .03). However, the occurrence of delayed or reduced dosages in the 2 groups was similar (20 of 131 [15.3%] in the nCRT group vs 12 of 130 [9.2%] in the nCT group; *P* = .14).

### Surgical Outcomes

Surgery was performed for 114 patients in the nCRT group and 108 patients in the nCT group (Figure). Among them, 112 patients in the nCRT group and 104 patients in the nCT group underwent esophagectomy. Two patients in the nCRT group underwent exploratory operations (1 owing to invasion of aorta and 1 owing to invasion of trachea), and 4 patients in the nCT group underwent exploratory operations (3 owing to invasion of aorta and 1 owing to invasion of trachea). Conversion to open thoracotomy occurred for 5 patients in the nCRT group and 3 in the nCT group. There were no significant differences between the 2 groups in surgical time, estimated blood loss, postoperative hospital stay, and retrieved lymph nodes (Table 1).

**Table 1.** Surgical Outcomes

Factor	nCRT group (n = 114)	nCT group (n = 108)	*P* value
Surgical approach, No. (%)			
MIE	107 (93.9)	101 (93.5)	.55
Conversion to open surgery	5 (4.4)	3 (2.8)	
Exploration	2 (1.8)	4 (3.7)	
Surgical time, mean (SD), min	265 (44)	256 (56)	.17
Estimated blood loss, mean (SD), mL	132 (64)	124 (53)	.32
Postoperative hospital stay, mean (SD), d	17.0 (12.0)	18.1 (13.5)	.53
Retrieved lymph nodes, mean (SD), No.^a^	24.9 (10.6)	25.4 (12.0)	.76

Abbreviations: MIE, minimally invasive esophagectomy; nCRT, neoadjuvant chemoradiotherapy; nCT, neoadjuvant chemotherapy.

^a^ Extensive mediastinal lymph node dissection and abdominal lymph node dissection was performed for all patients. Bilateral cervical lymph node dissection was added for 15 patients in nCRT group and 11 patients in nCT group.

### Morbidity and Mortality

The total postoperative complications rate was 47.4% in the nCRT group (54 of 114) and 42.6% in the nCT group (46 of 108) (Table 2).^22^ These rates were not significantly different between the groups (difference, 4.8%; 95% CI, −8.2% to 17.5%; *P* = .48). Eleven of 114 patients (9.6%) in the nCRT group and 12 of 108 patients (11.1%) in the nCT group had an anastomotic leak, with no statistically significant difference between groups (difference, –1.5%; 95% CI, −6.8% to 9.9%; *P* = .72). Moreover, according to the Clavien-Dindo classification, the distribution of severity was similar between the nCRT group and the nCT group (Clavien-Dindo classification IIIb or higher, 13 of 114 [11.4%] vs 11 of 108 [10.2%]; difference, 1.2%; 95% CI, −7.3% to 9.6%; *P* = .77). The rate of 90-day mortality was 3.5% in the nCRT group (4 of 114) and 2.8% in the nCT group (3 of 108) (*P* = .94). The causes of death were pulmonary function failure as a result of acute respiratory distress syndrome (1 patient in nCRT group), severe systemic inflammation caused by an anastomotic leak (1 patient in nCRT group and 1 patient in nCT group), massive hemorrhage of gastrointestinal tract (1 patient in nCT group), esophageal tracheal fistula (1 patient in nCRT group and 1 patient in nCT group), and superior mesenteric artery embolism (1 patient in nCRT group).

**Table 2.** Data on Morbidity and Mortality

Morbidity type or mortality	Patients, No. (%)		Between-group difference, RD (95% CI)^a^	*P* value
	nCRT group (n = 114)	nCT group (n = 108)		
All complications	54 (47.4)	46 (42.6)	4.8 (–8.2 to 17.5)	.48
Pneumonia	17 (14.9)	14 (13.0)	1.9 (–7.4 to 11.2)	.68
ARDS	3 (2.6)	2 (1.9)	0.8 (–7.4 to 11.2)	.95
Pneumothorax	2 (1.8)	1 (0.9)	0.8 (–4.2 to 5.8)	.96
Arrhythmia	5 (4.4)	3 (2.8)	1.6 (–4.1 to 7.4)	.78
Anastomotic leak	11 (9.6)	12 (11.1)	–1.5 (–6.8 to 9.9)	.72
Gastrointestinal bleeding	1 (0.9)	1 (0.9)	0 (–4.0 to 4.2)	.50
Liver function damage	2 (1.8)	0	1.8 (–1.7 to 6.5)	.50
Recurrent nerve injury	6 (5.3)	7 (6.5)	–1.2 (–5.4 to 8.1)	.70
Acute delirium	0	1 (0.9)	–0.9 (–2.4 to 5.1)	.98
Intrathoracic abscess	1 (0.9)	1 (0.9)	0 (–4.0 to 4.2)	.50
Wound infection	1 (0.9)	0	0.9 (–2.6 to 4.8)	.98
Generalized sepsis	1 (0.9)	0	0.9 (–2.6 to 4.8)	.98
Chylothorax	3 (2.6)	3 (2.8)	–0.1 (–5.0 to 5.5)	.73
Acute diaphragmatic hernia	1 (0.9)	0	0.9 (–2.6 to 4.8)	.98
Stroke (CVA)	0	1 (0.9)	–0.9 (–2.4 to 5.1)	.98
Clavien-Dindo grade				
I	27 (23.7)	25 (23.1)	0.5 (–10.6 to 11.6)	.93
II	5 (4.4)	3 (2.8)	1.6 (–4.1 to 7.4)	.78
IIIa	9 (7.9)	6 (5.6)	2.3 (–4.7 to 9.4)	.49
IIIb	8 (7.0)	9 (8.3)	–1.3 (–6.0 to 8.9)	.71
IVa	2 (1.8)	1 (0.9)	0.8 (–3.5 to 5.3)	.96
IVb	0	0	0	>.99
V	3 (2.6)	1 (0.9)	1.7 (–2.8 to 6.6)	.65
Clavien-Dindo grade IIIb or higher	13 (11.4)	11 (10.2)	1.2 (–7.3 to 9.6)	.77
90-d Postoperative mortality	4 (3.5)	3 (2.8)	0.7 (–4.8 to 6.2)	.94

Abbreviations: ARDS, acute respiratory distress syndrome; CVA, cerebrovascular accident; nCRT, neoadjuvant chemoradiotherapy; nCT, neoadjuvant chemotherapy; RD, rate difference.

^a^ Calculated using the Newcombe method.^22^

### Pathologic Outcome

The R0 resection rates were similar between the nCRT group and the nCT group (97.3% [109 of 112] vs 96.2% [100 of 104]; *P* = .92) (Table 3). Nevertheless, patients in the nCRT group had a better tumor regression grade (residual tumor, 0%: 40 of 112 [35.7%] vs 4 of 104 [3.8%]; *P* < .001), less lymph nodes involved (ypN0: 74 of 112 [66.1%] vs 48 of 104 [46.2%]; *P* = .03), and a better ypTNM stage than the nCT group (stage I: 58 of 112 [51.8%] vs 21 of 104 [20.2%]; *P* < .001).

**Table 3.** Pathologic Outcomes

Outcome	Patients, No. (%)		*P* value
	nCRT group (n = 112)	nCT group (n = 104)	
R0 resection	109 (97.3)	100 (96.2)	.92
Tumor regression grade			
1 (Residual tumor 0%)	40 (35.7)	4 (3.8)	<.001
2 (Residual tumor 1%-10%)	31 (27.7)	10 (9.6)	
3 (Residual tumor 11%-50%)	19 (17.0)	17 (16.3)	
4 (Residual tumor >50%)	22 (19.6)	73 (70.2)	
Lymph nodes involved			
ypN0	74 (66.1)	48 (46.2)	.03
ypN1	26 (23.2)	36 (34.6)	
ypN2	9 (8.0)	14 (13.5)	
ypN3	3 (2.7)	6 (5.8)	
ypTNM stage			
I^a^	58 (51.8)	21 (20.2)	<.001
II	11 (9.8)	21 (20.2)	
III	34 (30.4)	49 (47.1)	
IV^b^	9 (8.0)	13 (12.5)	

Abbreviations: nCRT, neoadjuvant chemoradiotherapy; nCT, neoadjuvant chemotherapy.

^a^ Including cases of ypT0N0M0: 31 (27.7%) in nCRT group and 3 (2.9%) in nCT group (*P* < .001).

^b^ The involved sites that were responsible for the stage IV disease were pleura invasion (3 in nCRT group and 5 in nCT group), pericardium invasion (2 in nCRT group and 1 in nCT group), and diaphragm crus invasion (1 in nCRT group and 1 in nCT group) as well as more than 7 positive lymph nodes (3 in nCRT group and 6 in nCT group).

### One-Year Follow-up of a Multicenter Randomized Clinical Trial

The 1-year overall survival rate using intention-to-treat analysis was 87.1% in the nCRT group (115 of 132) and 82.6% in the nCT group (109 of 132) (*P* = .30) (Table 4; eFigure in Supplement 2). Furthermore, deaths caused by tumor progression or recurrence were significantly less in the nCRT group than in the nCT group (9 of 132 [6.8%] vs 19 of 132 [14.4%]; *P* = .046); however, deaths from nontumor causes were similar (8 of 132 [6.1%] vs 4 of 132 [3.0%]; *P* = .24) (Table 4).

**Table 4.** Causes of Death Within 1 Year

Cause of death	Patients, No. (%)		*P* value
	nCRT group (n = 132)	nCT group (n = 132)	
Total death	17 (12.9)	23 (17.4)	.30
Tumor progression or recurrence	9 (6.8)	19 (14.4)	.046
Nontumor cause	8 (6.1)	4 (3.0)	.24
Surgical complication	5 (3.8)	3 (2.3)	.72
Serious adverse event	1 (0.8)	0	>.99
Other reason	2 (1.5)	1 (0.8)	>.99

Abbreviations: nCRT, neoadjuvant chemoradiotherapy; nCT, neoadjuvant chemotherapy.

## Discussion

This work is, to our knowledge, the first available well-designed multicenter randomized clinical trial with sufficient power to directly compare the safety and efficacy of nCRT vs nCT followed by surgery for locally advanced resectable ESCC. The initial result showed that the patients who underwent nCRT followed by MIE had no significantly added postoperative morbidities or increased mortality, but had a significantly better tumor regression grade and a higher rate of negative lymph nodes, as well as a better ypTNM stage, compared with those who underwent nCT. Thus, the regimen of nCRT based on paclitaxel and cisplatin followed by MIE seems to be feasible, safe, and effective for patients with locally advanced ESCC.

Currently, several important clinical trials have confirmed the role of nCRT therapy for patients with locally advanced esophageal cancer. In the CROSS trial,^23^ patients with esophageal cancer staging of cT1N1M0 or cT2 to T3N0 to 1M0 were enrolled, of whom 75% had adenocarcinoma, 23% had ESCC, and 2% had other subtypes. The nCRT group in the CROSS trial had a better R0 rate (92% vs 69%; *P* < .001), a lower rate of positive lymph nodes (31% vs 75%; *P* < .001), and longer overall survival (49.4 vs 24 months; *P* = .003) without significant postoperative morbidities or increased mortality compared with the nCT group. Moreover, the benefit of nCRT for survival was also confirmed in subgroups of patients with ESCC. In the Neoadjuvant Chemoradiotherapy Followed by Surgery Versus Surgery Alone for Locally Advanced Squamous Cell Carcinoma of the Esophagus (NEOCRTEC 5010) clinical trial enrolling patients with ESCC staging as T1 to 4N1M0 or T4N0M0, the nCRT group had a higher R0 resection rate (98.4% vs 91.2%; *P* = .002), a better median overall survival (100.1 vs 66.5 months; *P* = .03), and a prolonged disease-free survival (100.1 vs 41.7 months; *P* = .001) compared with patients undergoing surgery alone.^5^ On the other hand, the benefit of nCT followed by surgery has also been confirmed for locally advanced ESCC. In the JCOG 9907 trial, in which patients with clinical stage II or III (excluding T4) ESCC were enrolled, 5-year overall survival was higher among those who received nCT plus surgery than among those who received adjuvant chemotherapy (55% vs 43%; *P* = .04), and there were no remarkable differences in postoperative complications or mortality between the 2 groups.^6^

Nevertheless, the optimal modality for the treatment of locally advanced esophageal cancer remains unclear. At present, to our knowledge, there are only 3 clinical trials directly comparing nCRT with nCT. The Preoperative Therapy in Esophagogastric Adenocarcinoma Trial (POET),^8^ which was conducted from 2000 to 2005, enrolled 119 patients with clinical staging of T3 to 4NXM0, all of whom had esophagogastric junction adenocarcinoma. Hospital mortality was similar in the 2 groups (10.2% vs 3.8%; *P* = .26), and the morbidity information was not reported. The Neoadjuvant Chemotherapy Versus Radiochemotherapy for Cancer of the Esophagus or Cardia (NeoRes) trial,^9^ which was conducted from 2006 to 2013, enrolled 181 patients with clinical staging of T1 to 3NX (except T1N0), of which 73% were adenocarcinoma and 27% were ESCC. The results showed that the 2 groups had similar 30-day mortality (1% vs 0%; *P* > .99), 90-day mortality (8% vs 3%; *P* = .28), and total complications (55% vs 45%; *P* = .23); however, patients in the nCRT group were more likely to have Clavien-Dindo grade IIIb or higher (30% vs 17%; *P* = .05) as well as a higher mean comprehensive complication index (41 vs 31; *P* = .03). Moreover, the causes of death 1 year after randomization showed that 11 of 24 (45.8%) in the nCRT group and 3 of 20 (15.0%) in the nCT group (*P* = .04) died of treatment-related causes (severe adverse events during neoadjuvant therapy or postoperative complications). The clinical trial reported by Burmeister et al,^10^ which began in November 2000 and ceased in December 2006, enrolled 75 patients with clinical staging of T2 to 3N0 to 1M0, all of which were adenocarcinoma. There was no difference in the rates of surgical complication (23% vs 39%; *P* = .15) or 30-day surgical mortality (0% vs 0%; *P* > .99) between the 2 groups. The cases in these 3 trials were all or predominantly adenocarcinoma.

Therefore, to our knowledge, our clinical trial is the first one to directly compare nCRT with nCT for ESCC. No significant difference was found in the 2 neoadjuvant treatments for total complications, severe complications (Clavien-Dindo classification ≥IIIb), 90-day postoperative mortality, or treatment-related death 1 year after surgery. This result is somewhat different than the studies already mentioned. There are 2 reasons that could explain this distinction. One reason may be the differences in biological behavior between ESCC and adenocarcinoma. It is known that ESCC is different from esophageal adenocarcinoma in terms of the area of prevalence, tumor location, and lymph nodes involved, as well as surgical approaches and perioperative morbidities.^24,25^ In the NEOCRTEC 5010 trial that enrolled patients with ESCC, those in the nCRT group and those in the surgery alone group had similar rates of postoperative complications (57.8% vs 57.7%; *P* = .98) and peritreatment mortality (2.2% vs 0.4%; *P* = .21).^5^ Likewise, in the JCOG 9907 trial that enrolled patients with ESCC, there were no remarkable differences between those in the nCT group and those in the surgery alone group in terms of postoperative complications and mortality.^6^ Thus, it seems that patients with ESCC might have good acceptance and tolerance of nCRT and nCT.

Another reason that our results are somewhat different than the studies reported may be owing to the MIE used in our clinical trial instead of conventional open surgery. The high rate of postoperative mortality of patients who underwent neoadjuvant therapy followed by surgery in the previous trials may partly be due to the trauma caused by open esophagectomy. However, MIE could significantly decrease such trauma and decrease morbidity and mortality compared with open esophagectomy, which has been confirmed in the studies published.^13,14^ Moreover, several retrospective studies have suggested the safety and feasibility of MIE for locally advanced esophageal cancer after neoadjuvant therapy.^26,27^ Therefore, MIE could become the mainstream procedure for esophagectomy, although, to our knowledge, there are no other clinical trials available using this advanced technique.

As to the oncologic outcomes, the NeoRes trial reported a higher difference in R0 resection rates (87% vs 74%; *P* = .04) and pathologic complete response rates (28% vs 9%; *P* = .002) between the nCRT and nCT groups.^9^ POET reported similar R0 resection rates between nCRT and nCT groups (72% vs 69.5%; *P* = .91) but a much higher pathologic complete response rate in the nCRT group (15.6% vs 2.0%; *P* = .03).^8^ The trial reported by Burmeister et al^10^ found similar R0 resection rates between the nCRT and nCT groups (84.6% vs 80.5%; *P* = .89) and a better major histologic response rate (31% vs 8%; *P* = .01) and pathologic complete response rate in the nCRT group (13% vs 0%; *P* = .02). Although the different chemotherapy protocols used in these trials resulted in a difference in pathologic complete response rates, it seems that the advantage of nCRT vs nCT with regard to the pathologic complete response rate could be confirmed for esophageal adenocarcinoma. For ESCC, our trial first discovered that nCRT also had an advantage with regard to histolopathogic response. However, whether the better postoperative pathologic outcome would result in a survival benefit for patients with ESCC remains to be observed and determined by the 3- or 5-year survival rate in the follow-up.

### Limitations

This study has several limitations. Patients with poorer performance status and older patients were not recruited; therefore, the applicability of this therapy to these patients requires additional study. In addition, circumferential histologic margins were not reported in several centers in this trial, which could impact the accuracy of R0 rates. Moreover, the study was conducted for patients with ESCC, and whether these results are applicable for patients with esophageal or esophagogastric junction adenocarcinoma warrants additional investigation.

## Conclusions

This clinical trial showed that nCRT followed by MIE could result in a better histopathologic outcome, as well as similar morbidity and mortality rates, compared with nCT. It is worth observing the long-term survival benefit of nCRT vs nCT in the follow-up.

## Notes

Supplement 1. Trial Protocol

Supplement 2. eTable 1. Eligibility Criteria for Enrolling Patients eTable 2. The Baseline Clinical Characteristics of Patients eTable 3. Adverse Events During Neoadjuvant Therapy eFigure. The Overall Survival Curve of the Two Groups (Kaplan–Meier Plots)

Supplement 3. Data Sharing Statement
