# Supplementary material for: Morbidity and Mortality of Patients Who Underwent Minimally Invasive Esophagectomy After Neoadjuvant Chemoradiotherapy vs Neoadjuvant Chemotherapy for Locally Advanced Esophageal Squamous Cell Carcinoma: A Randomized Clinical Trial
Source: JAMA Surg. 2021 Mar 17;156(5):444–51. doi: 10.1001/jamasurg.2021.0133 (PMC7970392; doi:10.1001/jamasurg.2021.0133)
Supplement: Supplement 1. — Trial Protocol [file jamasurg-e210133-s001.pdf]

Trial Protocol and Statistical Analysis Plan

Version 2.0 (Final)

14th December 2016

CMISG1701 Trial: A Multicenter Prospective Randomized Phase III Clinical Trial Comparing Neoadjuvant Chemoradiotherapy to Neoadjuvant Chemotherapy followed by Minimally Invasive Esophagectomy in Patients with Locally Advanced Resectable Esophageal Squamous Cell Carcinoma（$cT_{3-4a}N_{0-1}M_0$）

CMISG1701 ESCC ($cT_{3-4a}N_{0-1}M_0$): nCRT + MIE VS. nCT + MIE

Principle Investigator (PI)

Prof.　Lijie Tan

Add: Department of Thoracic Surgery, Zhongshan Hospital, Fudan University, Fenglin Road 180, Shanghai 200032, China.

Tel: 86-21-64041990-2917

Fax: 86-21-64038477

Mobile: 86-13681972151

Email: tan.lijie@zs-hospital.sh.cn

Confidentiality Statement

The information contained in this clinical protocol is only available to the investigators, the Ethics Committee, and relevant agencies for review. Without an approval from the principal investigator (PI), any information shall not be informed to the third party irrelevant with this study.

Summary

Protocol Title	CMISG1701 Trial: A Multicenter Prospective Randomized Phase III Clinical Trial Comparing Neoadjuvant Chemoradiotherapy (nCRT) to Neoadjuvant Chemotherapy (nCT) followed by Minimally Invasive Esophagectomy (MIE) in Patients with Locally Advanced Resectable Esophageal Squamous Cell Carcinoma (cT3-4aN0-1M0)
Protocol Version	Version 2.0
Sponsor	Lijie Tan
Research Center Number	10
Research Centers	1 Zhongshan Hospital, Fudan University, Shanghai, China;<br>2 The First Affiliated Hospital of Chongqing Medical University, Chongqing, China;<br>3 Tianjin Medical University Cancer Institute and Hospital, Tianjin, China;<br>4 Shanghai Chest Hospital, Shanghai Jiao Tong University, Shanghai，China;<br>5 The First Affiliated Hospital of Wenzhou Medical University, Wenzhou, China;<br>6 Heping Hospital Affiliated to Changzhi Medical College, Changzhi, Shanxi, China;<br>7 Fujian Medical University Union Hospital, Fuzhou, Fujian, China;<br>8 Union Hospital, Tongji Medical College, Huazhong University of

	Science and Technology, Wuhan, Hubei, China;<br><br>9 Ruijin Hospital, Shanghai JJiao Tong University School of Medicine, Shanghai, China;<br><br>10 Peking University Cancer Hospital and Institute, Peking University School of Oncology, Beijing, China.
Indications	Patients with Locally Advanced Resectable Esophageal Squamous Cell Carcinoma (cT3-4aN0-1M0)
Research Purpose	This study aims to determine the advantage of nCRT plus MIE compared with nCT plus MIE for patients with locally advanced resectable ESCC (cT3-4aN0-1M0).
Research Design	prospective, multicenter, randomized, controlled, open, phase III trial.
Case Grouping	• Group A (study group): nCRT plus MIE<br><br>• Group B (control group): nCT plus MIE
Sample Size	The sample size calculations are based on the primary outcome overall survival. From our own experience, the 3-year overall survival rate is 72.7% and 47.1% for patients in nCRT group and in nCT group without differences in mortality, respectively. Therefore, the total number of sample size is 264, which is based on the intention of showing a benefit of nCRT arm (arm A) over the other arm (arm B) in the primary end point of 20% with a one-sided type I error of 5% and a power of 90% as well as 15% drop out before surgery or lost to follow up according to Power Analysis and Sample Size (PASS). Thus, 134 patients were enrolled in each arm with the balance of age, N stage and trial center according to 1:1 randomized allocation. The sample size will ensure sufficient power to demonstrate an overall

	survival advantage of nCRT over nCT by the end of the trial.
Inclusion Criteria	1. Aged 18-75 years;<br>2. Histologically-confirmed squamous cell carcinoma of the esophagus;<br>3. Tumors of the esophagus are located in the thoracic cavity;<br>4. Pre-treatment stage as cT3-4aN0-1M0 (AJCC/UICC 7th Edition) (In case of stage cT4a, curative resectability has to be explicitly verified by the local surgical investigator prior to randomization);<br>5. Eastern Cooperative Oncology Group (ECOG) performance status 0–1;<br>6. Adequate cardiac function. All patients should perform ECG, and those with a cardiac history or ECG abnormality should perform echocardiography with the left ventricular ejection fraction > 50 %.<br>7. Adequate respiratory function with FEV1≥1.2L, FEV1%≥50% and DLCO≥50% shown in pulmonary function tests.<br>8. Adequate bone marrow function (White Blood Cells >4x10^9 /L; Neutrophil >2.0×10^9 /L; Hemoglobin > 90 g/L; platelets>100x10^9 /L);<br>9. Adequate liver function (Total bilirubin <1.5x Upper Level of Normal (ULN); Aspartate transaminase(AST) and Alanine transaminase (ALT) <1.5x ULN);<br>10. Adequate renal function (Glomerular filtration rate (CCr) >60 ml/min; serum creatinine (SCr) ≤120 μmol/L)；

	11. The patient has provided written informed consent and is able to understand and comply with the study.
Exclusion Criteria	1. Patients with non-squamous cell carcinoma histology;<br>2. Patients with advanced inoperable or metastatic esophageal cancer;<br>3. Pre-treatment stage as cT1-2N0-1M0 (AJCC/UICC 7th Edition);<br>4. Pre-treatment stage as cN2-3 or cT4b (non-curatively-resectable verified by the local surgical investigator, AJCC/UICC 7th Edition);<br>5. Patients with another previous or current malignant disease which is likely to interfere with treatment or the assessment of response in the judgement of the local surgical investigator.<br>6. Any patient with a significant medical condition which is thought unlikely to tolerate the therapies. Such as cardiac disease (e.g. symptomatic coronary artery disease or myocardial infarction within last 12 months), clinically-significant lung disease, clinically-significant bone marrow, liver, renal function disorder;<br>7. Pregnant or lactating women and fertile women who will not be using contraception during the trial;<br>8. Allergy to any drugs;<br>9. Participation in another intervention clinical trial with interference to the chemotherapeutic or chemoradiotherapeutic intervention during this study or during the last 30 days prior to informed consent;<br>10. Expected lack of compliance with the protocol.

Withdraw Criteria	1. Confirmed that it is unable to do resection due to the disease progression after neoadjuvant treatment;<br><br>2. Patients requiring simultaneous surgical treatment for other diseases;<br><br>3. Sudden severe comorbidities in the perioperative period (intolerable surgery or anesthesia), which are unsuitable or unable to implement the treatment protocol of this study as scheduled;<br><br>4. Patients are confirmed to require emergency surgery according to the condition changes verified by attending doctors after being enrolled in this study;<br><br>5. Patients are voluntary to quit or discontinue treatment due to personal reasons in any stage after being enrolled in this study;<br><br>6. Treatment that proved to violate the study protocol.
Intervention	• Group A: Neoadjuvant chemoradiation + MIE<br><br>• Group B: Neoadjuvant chemotherapy + MIE
Endpoint	Primary Endpoint:<br><br>• Overall survival time in the intent-to-treat population, which ends with the date of death of any causes since the date of randomization assessed up to 36 months. For patients alive at study closure, the survival time will be censored at time of last known survival status.<br><br>Secondary Endpoint:<br><br>1. Progression-free survival (PFS) time: It is defined as the time from the date of randomization to the date of first recurrence/progression

	(local, regional or distant) or death assessed up to 36 months. Progression is examined by computed tomography (CT), positron emission tomography-computed tomography (PET-CT) and/or upper endoscopy.<br><br>2. Recurrence-free survival (RFS) time: It is defined as the time from the date of surgery to the date of first recurrence (local, regional or distant) or death assessed up to 36 months. Recurrence is examined by CT, PET-CT and/or upper endoscopy.<br><br>3. Postoperative pathologic stage:<br><br>(1) Pathological complete response rate (pCR): Pathological complete response rate (pCR) is to be assessed in the resected specimen following neoadjuvant therapy using standardized work up of the resection specimen in the pathology department and standardized histological criteria for tumor regression grading. The degree of histomorphologic regression is clarified into four categories as follows: grade 1, no evidence of vital residual tumor cells (pathological complete response); grade 2, less than 10% vital residual tumor cells; grade 3, 10 to 50%; and grade 4, more than 50% according to previous report.<br><br>(2) R0 resection rate: No vital tumor is presented at the proximal, distal, or circumferential resection margin, then it is considered to be R0 resection. If a vital tumor is shown at 1 mm or less from the proximal, distal, or circumferential resection margin, it is considered to be microscopically positive (R1).<br><br>(3) Positive lymph nodes' number: According to pathological reports, record the number of positive lymph nodes.

	(4) Pathological TNM stage.<br>4. Treatment related complications: Record the data according to International Consensus of Esophagectomy Complications Consensus Group (ECCG). Chemoradiation/chemotherapy-related toxicities during preoperative time are collected according to CTCAE version 4.03;<br>5. Postoperative mortality: 30-day postoperative mortality;<br>6. Quality of life (QOL): QOL is respectively evaluated at randomization, 4 weeks after neoadjuvant therapy and 1 month, 4 month, 7 month and yearly after surgery among patients by using the European Organization for Research and Treatment of Cancer Quality of Life Questionnaire C-30 (EORTC QLQ-C30) and EORTC QLQ-OES18, it is assessed up to 36 months.
Statistical considerations	● Statistical software: SAS statistical software.<br>● Descriptive statistics:<br>■ Continuous data: number of cases (number of missing cases), mean, median, standard deviation, P25, P75, minimum and maximum;<br>■ Categorical data: frequency and the corresponding percentages. For primary safety endpoint, calculate the 95% CI in addition to the percentage.<br>● Statistical inference: unless otherwise specified, the two-sided P≤0.05 indicates statistically significant differences between the two groups.<br>■ Statistical analysis for primary endpoint: the 3-year overall

	survival rates in the two treatment arms will be calculated by the Kaplan-Meier method and compared by the log rank test. The Cox proportional hazard model will be used to evaluate the survival-independent factors.<br>■ Statistical analysis for baseline variables and secondary endpoints: continuous variables were examined by independent sample t-test or Wilcoxon rank-sum test, and categorical variables were compared by Pearson chi-square test, Fisher’s exact test or CMH chi-square test as appropriate.<br>■ Analysis of withdrawn patients: the number of patients who are enrolled, withdrawn, removed, completed, and number of every analysis set will be listed.

1 Background

Esophageal cancer is one of the most common digestive tract cancers worldwide[1]. It is reported the incidence and death rate of esophageal cancer in China is the highest in the world, with its morbidity expecting to ascend to the third place and its mortality expecting to rise to the fourth position according to the Cancer Statistics in China, 2015[2]. Notably, esophageal squamous cell carcinoma (ESCC) accounts for more than 90% of all cases in China. Traditional curative esophagectomy still plays an important role in the treatment of esophageal cancer, however, curative resection alone often accompanies with high recurrence and metastasis rates, low 3 and 5-year overall survival, especially in patients with locally advanced resectable esophageal cancer($cT_{3-4a}N_{0-1}M_0$)[1]. Therefore, multimodality therapy has been developed in order to improve the prognosis.

Neoadjuvant therapy has been explored for many years in western countries and Japan, and proved to get survival benefit, especially for locally advanced esophageal cancer. The CROSS trial performed by van Hagen et al[3] was acknowledged as the most representative one among studies comparing neoadjuvant chemoradiation (nCRT) plus surgery versus surgery alone for patients with adenocarcinoma or squamous cell carcinoma of the esophagus. Patients with esophageal cancer staging as $cT_1N_1M_0$ or $cT_{2-3}N_{0-1}M_0$ were enrolled in the study, and it showed better R0 rate (92% vs 69%, P<0.001), lower node-positive rate (31% vs 75%, P<0.001) and longer overall survival (49.4 vs 24 months, P=0.003) in the nCRT group without significant postoperative morbidities and mortalities. The benefit of nCRT on survival was also confirmed in subgroups with ESCC. Nowadays, many studies [4-7] verified the fact that a significant overall survival benefit was achieved with nCRT plus surgery compared to surgery alone for patients with ESCC. However, accumulating evidence suggested that a significant level of toxicity resulted from nCRT for ESCC. Specifically, FFCD 9901 trial[8] indicated nCRT resulted in significant postoperative

mortality (11.1% vs 3.4%, P =0.049) without benefits of 3-year overall survival rate (47.5% vs 53.0%, P =0.94), which was stopped for anticipated futility. In addition, Kumagai et al[9] summarized 23 RCTs about neoadjuvant therapy via meta-analysis, and it also demonstrated nCRT plus surgery was associated with a significantly higher risk of total postoperative mortality (HR=1.95, P=0.032) and treatment-related mortality (RR 1·97, P=0·030) compared with surgery alone. Thereafter, nCRT has not been perceived as a safe approach, while neoadjuvant chemotherapy (nCT), which showed an improved survival rate compared with surgery alone, has been demonstrated safe by many studies [6, 9-11] and is being applied as an standard approach for treatment of ESCC.

With the development of techniques and innovation of instruments, minimally invasive esophagectomy (MIE) is introduced into practice worldwide. Due to less trauma, fewer complications as well as similar curative effect, MIE tends to take the place of traditional open esophagectomy and becomes the mainstream procedure[12-14]. There is no doubt that higher postoperative mortality of nCRT results partly from the huge trauma caused by open esophagectomy. Therefore, it is worthwhile to investigate whether MIE could lower the risk of mortality in nCRT approach. Some retrospective studies reported MIE was an acceptable surgical therapy for advanced-stage esophageal malignancies after nCRT without evidence of increased morbidity or mortality[15, 16]. As far as I can see, there are only two prospective randomized studies exploring the outcomes between nCRT plus surgery and nCT plus surgery, which showed higher complete response rate, lower recurrence rate and improved 3-year overall without increased mortality in nCRT group, but it should be pointed that these two studies were confined to esophageal adenocarcinoma[17, 18].

As is known, there are no any studies concentrating on comparing nCRT to nCT followed by MIE in patients with locally advanced resectable ESCC ($cT_{3\text{-}4a}N_{0\text{-}1}M_0$) so far. Our preliminary work confirmed nCRT followed by MIE was a safe and effective

option to treat locally advanced resectable ESCC ($cT_{3-4a}N_{0-1}M_0$) compared with nCT, of which initial results showed higher complete response rate, lower node-positive rate and longer survival time without increased morbidity and mortality (data not published). Hereby, we launch this multicenter prospective randomized phase III clinical trial aiming at investigating and verifying the advantage of nCRT plus MIE in treatment of ESCC. This is the only comparative analysis on nCRT versus nCT in patients with locally advanced resectable ESCC ($cT_{3-4a}N_{0-1}M_0$).

According to the given evidence, a survival benefit of nCRT or nCT plus surgery over surgery alone for locally advanced resectable ESCC has been proved in many RCT studies, but the potential higher risk of postoperative mortality imposes restrictions on nCRT's application in treating ESCC. As is known to all, MIE has significant advantages in decreasing postoperative morbidity and mortality compared with open surgery and has been proved to be feasible in nCRT, however, we have no idea about whether it could reduce mortality when combined with neoadjuvant therapy. As neoadjuvant therapy plus MIE is extensively and successfully applied in clinical practice in patients with ESCC and no RCTs have concentrated on comparing the outcomes between nCRT and nCT followed by MIE, there is a clear need to obtain evidence concerning the value of nCRT plus MIE in patients with locally advanced resectable ESCC ($cT_{3-4a}N_{0-1}M_0$) from a multicenter RCT.

2 Objective

The objective of this trial is to determine the safety and efficacy of nCRT plus MIE compared with nCT plus MIE for locally advanced resectable esophageal squamous cell carcinoma (clinical staged cT3-4aN0-1M0)

3 Design

CMISG1701 is a prospective, multicenter, randomized, controlled, open, phase III trial.

3.1 Multicenter

Ten high-volume centers from Shanghai, Beijing, Tianjin, Chongqing, Fuzhou, Wenzhou, and Changzhi participated in this study.

3.2 Grouping and Control

Group A: Neoadjuvant chemoradiation + MIE

Group B: Neoadjuvant chemotherapy + MIE

3.3 Sample Size

The sample size calculations are based on the primary outcome overall survival. From our own experience, the 3-year overall survival rate is 72.7% and 47.1% for patients in nCRT group and in nCT group without differences in mortality, respectively. Therefore, the total number of sample size is 264, which is based on the intention of showing a benefit of nCRT arm (arm A) over the other arm (arm B) in the primary end point of 20% with a one-sided type I error of 5% and a power of 90% as well as 15% drop out before surgery or lost to follow up according to Power Analysis

and Sample Size (PASS). Thus, 134 patients were enrolled in each arm with the balance of age, N stage and trial center according to 1:1 randomized allocation. The sample size will ensure sufficient power to demonstrate an overall survival advantage of nCRT over nCT by the end of the trial.

3.4 Randomization

In this study, the central dynamic, stratified randomization method is adopted, and the factors including age, gender, and investigators, are considered. After each case is enrolled, the research center will arrange the research assistant to send the information of included cases (age, gender) to the data center through email, telephone, and SMS, etc. After analyzing the case information by the center randomization department, the case grouping will be determined.

3.5 Blind Method

Open design is adopted in this study.

4 Research Subjects

Patients that meet all the inclusion criteria and are beyond any one of exclusion criteria are eligible for this study.

4.1 Inclusion Criteria

1. Aged 18-75 years;
2. Histologically-confirmed squamous cell carcinoma of the esophagus;
3. Tumors of the esophagus are located in the thoracic cavity;
4. Pre-treatment stage as cT3-4aN0-1M0 (AJCC/UICC 7th Edition) (In case of stage cT4a, curative resectability has to be explicitly verified by the local surgical investigator prior to randomization);
5. Eastern Cooperative Oncology Group (ECOG) performance status 0–1;
6. Adequate cardiac function. All patients should perform ECG, and those with a cardiac history or ECG abnormality should perform echocardiography with the left ventricular ejection fraction > 50 %.
7. Adequate respiratory function with FEV1≥1.2L, FEV1%≥50% and DLCO≥50% shown in pulmonary function tests.
8. Adequate bone marrow function (White Blood Cells >4x10^9 /L; Neutrophil >2.0×10^9 /L; Hemoglobin > 90 g/L; platelets>100x10^9 /L);
9. Adequate liver function (Total bilirubin <1.5x Upper Level of Normal (ULN); Aspartate transaminase(AST) and Alanine transaminase (ALT) <1.5x ULN);
10. Adequate renal function (Glomerular filtration rate (CCr) >60 ml/min; serum creatinine (SCr) ≤120 μmol/L)；
11. The patient has provided written informed consent and is able to understand and comply with the study.

4.2 Exclusion Criteria

1. Patients with non-squamous cell carcinoma histology;

2. Patients with advanced inoperable or metastatic esophageal cancer;

3. Pre-treatment stage as cT1-2N0-1M0 (AJCC/UICC 7th Edition);

4. Pre-treatment stage as cN2-3 or cT4b (non-curatively-resectable verified by the local surgical investigator, AJCC/UICC 7th Edition);

5. Patients with another previous or current malignant disease which is likely to interfere with treatment or the assessment of response in the judgement of the local surgical investigator.

6. Any patient with a significant medical condition which is thought unlikely to tolerate the therapies. Such as cardiac disease (e.g. symptomatic coronary artery disease or myocardial infarction within last 12 months), clinically-significant lung disease, clinically-significant bone marrow, liver, renal function disorder;

7. Pregnant or lactating women and fertile women who will not be using contraception during the trial;

8. Allergy to any drugs;

9. Participation in another intervention clinical trial with interference to the chemotherapeutic or chemoradiotherapeutic intervention during this study or during the last 30 days prior to informed consent;

10. Expected lack of compliance with the protocol.

4.3 Withdraw Criteria

1. Confirmed that it is unable to do resection due to the disease progression after neoadjuvant treatment;

2. Patients requiring simultaneous surgical treatment for other diseases;

3. Sudden severe comorbidities in the perioperative period (intolerable surgery or anesthesia), which are unsuitable or unable to implement the treatment protocol of this study as scheduled;

4. Patients are confirmed to require emergency surgery according to the condition changes verified by attending doctors after being enrolled in this study;

5. Patients are voluntary to quit or discontinue treatment due to personal reasons in any stage after being enrolled in this study;

6. Treatment that proved to violate the study protocol.

5 Endpoints

5.1 Primary Endpoint

Overall survival time in the intent-to-treat population, which ends with the date of death of any causes since the date of randomization assessed up to 36 months. For patients alive at study closure, the survival time will be censored at time of last known survival status.

5.2 Secondary Endpoint

1. Progression-free survival (PFS) time: It is defined as the time from the date of randomization to the date of first recurrence/progression (local, regional or distant) or death assessed up to 36 months. Progression is examined by computed tomography (CT), positron emission tomography-computed tomography (PET-CT) and/or upper endoscopy.

2. Recurrence-free survival (RFS) time: It is defined as the time from the date of surgery to the date of first recurrence (local, regional or distant) or death assessed up to 36 months. Recurrence is examined by CT, PET-CT and/or upper endoscopy.

3. Postoperative pathologic stage:

(1) Pathological complete response rate (pCR): Pathological complete response rate (pCR) is to be assessed in the resected specimen following neoadjuvant therapy using standardized work up of the resection specimen in the pathology department and standardized histological criteria for tumor regression grading. The degree of histomorphologic regression is clarified into four categories as follows: grade 1, no evidence of vital residual tumor cells (pathological complete response); grade 2, less than 10% vital residual tumor cells; grade 3, 10 to 50%; and grade 4, more than 50% according to previous report.

(2) R0 resection rate: No vital tumor is presented at the proximal, distal, or circumferential resection margin, then it is considered to be R0 resection. If a vital tumor is shown at 1 mm or less from the proximal, distal, or circumferential resection margin, it is considered to be microscopically positive (R1).

(3) Positive lymph nodes' number: According to pathological reports, record the number of positive lymph nodes.

(4) Postoperative TNM stage.

4. Treatment related complications: Record the data according to International Consensus of Esophagectomy Complications Consensus Group (ECCG). Chemoradiation/chemotherapy-related toxicities during preoperative time are collected according to CTCAE version 4.03;

5. Postoperative mortality: 30-day postoperative mortality;

6. Quality of life(QOL): QOL is respectively evaluated at randomization, 4 weeks

after neoadjuvant therapy and 1 month, 4 month, 7 month and yearly after surgery among patients by using the European Organization for Research and Treatment of Cancer Quality of Life Questionnaire C-30 (EORTC QLQ-C30) and EORTC QLQ-OES18, it is assessed up to 36 months.

6 Diagnostic Criteria

AJCC-7th TNM tumor staging system is adopted in this study;

7 Qualification of the Responsible Surgeons

The responsible surgeons should meet the following qualifications:

- Completing at least 100 cases of MIE respectively;
- Passing the blind review of surgery video.

8 Standard Operation Procedures (SOP)

Treatment Schedule

**Treatment Phase**	**Screening**	**Neoadjuvant treatment**	**Preoperative evaluation**	**Operation**	**Follow up**
Time point	<14 days before Randomization	Week 1-5<br>At day 1 of each week	Within 3-5 weeks after neoadjuvant treatment	Day of hospital discharge from Surgery	Starting 1 month after surgery, every 3/6 months*
Items	**(Vs)**	**(V_n)**	**(Vr)**	**(Vs)**	**(F1--x)**
Informed consent	x				
Inclusion/exclusion	x				
Demography[i]	x				
Medical history	x				
Vital sign(P, R, T, BP)	x	x	x	x	
Physical examination[ii]	x		x		x
Body weight	x	x	x	x	x
ECOG performance score	x				
CT thorax/abdominal	x[iii]		x		x[iv]
Endoscopic ultrasound	x		x		x
Upper GI endoscopy	x				
gastroscopy	x				x[v]
Histopathology report	x			x	
Tissue specimen[vi]	x			x	

**Treatment Phase**	**Screening**	**Neoadjuvant treatment**	**Preoperative evaluation**	**Operation**	**Follow up**
Time point	<14 days before Randomization	Week 1-5<br>At day 1 of each week	Within 3-5 weeks after neoadjuvant treatment	Day of hospital<br>discharge from Surgery	Starting 1 month after surgery, every<br>3/6 months*
Items	**(Vs)**	**(V_n)**	**(Vr)**	**(Vs)**	**(F1--x)**
ECG[vii]	x		x		
Pulmonary function	x		x		
Blood routine and biochemistry [viii]	x	x	x	x	
Laboratory Infection[ix]	x				
Laboratory Coagulation[x]	x		x		
Tumor biomarker[xi]	x		x		x
Pregnancy test (only women)	x				
Randomization	x				
Blood sample[xii]	x			x	×
Adverse events and complications [xiii]	x				
Concomitant medication[xiv]	x				
Quality of life (EORTC QLQ-C30, OES18,)[xv]	x		x	x	x

----

* The first follow-up visit is performed 1 months after surgery. From then on, follow-up visits are carried out every 3 months (+/- 7days) in the first two years of follow-up and every 6 months (+/- 7days) from the third year after treatment until the end of follow-up (min. 3 years).

Demography includes sex, age, height, race, ethnicity, job category, allergy and so on

Physical examination includes, but is not limited to, cardiovascular, gastrointestinal, hepatobiliary, respiratory, musculoskeletal, skin, neurological, genitourinary/renal and other organ systems.

Not older than 14 days before date of randomisation，and it must be contrast-enhanced CT. If suspected to be T_{4b} stage, multiregional lymph node metastases or distant metastases, PET-CT or endoscopic ultrasound (EUS) (selectable) is performed to ensure pre-treatment cTNM stage.

Contrast-enhanced CT of Thorax/Abdomen is carried out regularly at follow-up visits. Further diagnostic investigations, including PET-CT, Upper endoscopy are performed only if suspected to be recurrence or metastasis at the discretion of the investigator/treating physician.

Gastroscopy is performed once a year.

Representative blocks from the initial biopsy and the operative specimen will be requested from the reporting pathologists.

ECG must be performed during screening and preoperative. Patients with a cardiac history should have echocardiography, further cardiac examinations can be performed if necessary to exclude contraindication.

Blood routine includes hemoglobin, total red count, total white blood count, platelet count, and a differential white count including neutrophils, lymphocytes, monocytes, eosinophils and basophils. Biochemistry includes (but is not limited to) AST, ALT, total bilirubin, blood glucose, serum creatinine, sodium, potassium.

HBV、HCV、HIV serological examinations

Coagulation includes PT, PTT 和 INR。

Tumor biomarker includes CEA, CA19-9, CA125, CYFRA21-1 and SCC.

Two blood samples are collected for translational research before treatment, before surgery, 4 months after surgery and the time of recurrence or metastasis, respectively.

The AE reporting period for this trial begins after first intake of medication within the study and until 8 months after randomisation. All adverse events have to be documented in the CRF.

Concomitant medication must be available in the source data and don`t be captured in the CRF.

Quality of life is recorded before treatment, 4 weeks after neoadjuvant therapy and 1 month, 4 month, 7 month, yearly after surgery, respectively.

8.1 Case Selection

When admitted to hospital, the potential patients who are meeting all the inclusion criteria and are beyond any one of exclusion criteria are selected.

8.1.1 Assessment Item

The clinical examination results that got between hospital admission and study enrollment (usually 1 week) are determined as the baseline data. These data must include:

1) General status: height, weight, ECOG performance score, ASA score;
2) Peripheral venous blood: Hb, RBC, WBC, LYM, NEU, NEU%, PLT;
3) Blood biochemical indexes: ALB, prealbumin, TBil, DBil, AST, ALT, Cr, BUN, BG, CRP, HbA1c, Glycated albumin;
4) Serum tumor markers: CEA, SCC;
5) Imaging examinations, including thoracoabdominal enhanced computed tomography, cervical ultrasonography, endoscopic ultrasonography (performed as possibly) and positron emission tomography (optional when necessary)
6) Standard 12-lead electrocardiogram;
7) Pulmonary function examination: FEV1, FVC.

8.1.2 Selection Application

Before enrollment in this study, the research assistant of each research center should fill in the [Eligibility Application Form] for patients that meet all the inclusion criteria and are beyond any one of exclusion criteria and then send it to the PI research team through e-mail or fax for reviewing whether the patients are eligible.

8.1.3 Eligibility Consulting

Contact Information and Working Hours of Research Committee:

Add: Research Committee of Esophageal Cancer Treatment, Zhongshan Hospital, Fudan University

Tel: 021-64041990 -2917

Working Hours: Monday to Friday, 9:00 to 17:00 (except weekends and holidays)

Contact Information:

Lijie Tan

Add: Department of Thoracic Surgery, Zhongshan Hospital, Fudan University, Fenglin Road 180, Xuhui District, Shanghai.

Code: 200032

Tel: 021-64041990 -2917

Mobile: 13681972151

E-mail: tan.lijie@zs-hospital.sh.cn

Hao Wang

Add: Department of Thoracic Surgery, Zhongshan Hospital, Fudan University, Fenglin Road 180. Xuhui District, Shanghai.

Code: 200032

Mobile: 13816051785

E-mail: wang.hao@zs-hospital.sh.cn

8.1.4 Attentions

(1) The application and confirmation of eligibility should be completed preoperatively.

(2) [Eligibility Application Form] must be completely filled; otherwise, it will not be accepted.

(3) After accredited by the Research Committe, the case should be numbered (Baseline Number, BN), and the [eligibility confirmation notice] should be emailed to the applicant.

(4) The research assistant of each center is responsible for the [eligibility confirmation notice] keeping.

(5) Once selected for registration, the content of the [eligibility application form] will be entered into the database, and the eligibility is not allowed to be artificially canceled (the relevant information cannot be deleted from the database), unless the patient declines the information to be used in this study.

(6) The data center will reject any repeatedly registered information. If it happens, the first registered data will be used (first BN).

(7) In case of repeat selection or incorrect registration, the research assistant of each research center should contact the Research Committee and record it.

8.2 Written Informed Consent

The written informed consent is provided by the patient after comprehensively

understanding of the trial.

8.3 Randomized Grouping

The patients were randomly assigned in a 1:1 allocation ratio to receive nCRT followed by surgery (nCRT group) or nCT followed by surgery (nCT group) and were stratified according to coordinating centers. Random was assigned by the computer-generated random system in the Biomedical Statistics Center, Fudan University. Each assignment was generated after the completion of this patient registration in the random system online.

8.4 Neoadjuvant Treatment

8.4.1 Neoadjuvant chemoradiotherapy

On days 1, 8, 15 and 22, paclitaxel at a dose of 50mg/m^2 and cisplatin at a dose of 25mg/m^2 of body-surface area will be administered by intravenous drip infusion. A total dose of 40Gy will be administered in 20 fractions of 2Gy, five fractions per week, starting at the first day of the chemotherapy. All patients will be treated by means of external beam radiation.

8.4.2 Neoadjuvant chemotherapy

The nCT group consists of two cycles of preoperative chemotherapy before surgery. The regimen is paclitaxel at a dose of 135mg/m^2 and cisplatin at a dose of 75mg/m^2 on day 1 by intravenous drip infusion. And the second cycle will be given after 3 weeks.

8.5 Assessments during the Neoadjuvant Treatment

The patients will be closely monitored for toxic effects of chemotherapy with the use of the National Cancer Institute's Common Terminology Criteria for Adverse Events, version 5.0. Vital sign, body weight, description of discomfort symptoms and standard laboratory tests (blood routine, blood biochemistry) will be obtained and recorded weekly before and during neoadjuvant therapy period, which assessed the toxicity of preoperative therapy. After 4 weeks of neoadjuvant therapy, a CT (or PET-CT) scan of thorax and abdomen and ultrasound of the neck will be performed to re-stage of the tumor.

8.6 Surgical Procedure

After 4-8 weeks of neoadjuvant therapy, MIE will be performed. The procedure in details is referred in previous article[19-21]. To achieve an accurate ypTNM stage, the extent of lymphadenectomy demands resecting radically. Dissected lymph nodes

were classified according to lymph node stations adopted by the Japanese Classification[22]. The dissected nodes in thoracic cavity should include the upper paraesophageal (no.105), paratracheal (no.106r and 106tb), subcarinal (no.107), middle paraesophageal (no.108), bilateral hilar lymph nodes (no.109), lower paraesophageal (no.110), posterior mediastinal lymph nodes (no.111), and diaphragmatic (no.112) ones. The dissected abdominal nodes should include the nodes lateral to the paracardia, lesser curvature, greater curvature, left gastric, common hepatic, splenic, and celiac stations. If neoplasm is located at upper mediastinum, cervical nodes in the cervical paraesophageal (no.101) and supraclavicular regions (no.104) should be dissected.

8.7 Observation Items during the Operation

The research assistant after operation should record the specific items:

(1) Name of doctor in charge;

(2) Operation starting time (min), Operation finishing time (min);

(3) Operation type, extent of lymphadenectomy, reconstruction method;

(4) Incision length (cm), number of trocars;

(5) Whether the MIE is transferred to open surgery and reasons;

(6) Estimated blood loss during operation (ml);

(7) Volume of blood transfusion (defined as transfusion of red cell suspension or

plasma, ml);

(8) Tumor position;

(9) Tumor size;

(10) Invasion depth, distant metastasis (position);

(11) Proximal resection margin length (mm), distal resection margin length (mm), radical degree (R0/R1/R2);

(12) Intraoperative complications

(13) Intraoperative death: regardless of any reason.

8.8 Postoperative Management

8.8.1 Fluid Infusion and Nutritional Support

- Postoperative fluid infusion (including glucose, insulin, electrolytes, vitamins etc.) or nutritional support (enteral/parenteral) is performed according to the experience of the doctor in charge and clinical routines, which is not specified in this study.
- After oral feeding, fluid infusion/ nutritional support should gradually reduce until stop.

8.8.2 Rehabilitation Management

- Management of incision, chest tube, cervical drainage-tube, and abdominal cavity drainage-tube: Following the clinical routines.
- Recovery eating time and transition strategies of diet: Following the clinical

routines.

8.8.3 Discharge Standard

- No postoperative complications, meeting “body temperature is less than 37”, “the pain can be tolerated”, and “starting oral intake for more than 2 days”, a patient can be arranged for discharge, which should be recorded in the CRF.

8.8.4 Postoperative Observation Items

- Definition of postoperative “n days”: One day from 0:00 to 24:00. The time frame from the end of surgery to 24:00 of the surgery day is defined as “postoperative 0 day”; the next day from 0:00 to 24:00 is “postoperative 1 day”, and so on.
- From postoperative 1 day to discharge day, the research assistant should timely record the items. The observation items include:

(1) Pathological Results:

- Surgical outcomes (R0/R1/R2);
- Histological type of primary lesion;
- Depth of esophageal wall invasion;
- Histological grade (G1/G2/G3/G4/GX);
- Lymphovascular invasion;
- Total number of retrieved lymph nodes, number of lymph nodes in each group, number of lymph node metastasis in each group, and the total number of lymph

node metastasis;

(2) Early postoperative complications:

- Time frame: 30 days after operation (postoperative hospital stay ≤ 30 days) or operation to first discharge from hospital (postoperative hospital stay > 30 days).
- Observation items:

①Surgery-related complications：Wound complications (infection, effusion, dehiscence, poor healing, etc.), active bleeding, anastomotic stenosis, intestinal fistula, pancreatic fistula, chylous fistula, abscess formation, intestinal paralysis, intestinal obstruction, cholecystitis, pancreatitis, etc.

②System-related complications: Pneumonia, pleural effusion, pulmonary embolism, cardio-cerebrovascular complications (including thrombosis and embolism), deep venous thrombosis, urinary tract complications, catheter-related complications, etc.

- Classification of Surgical Complications (Clavien-Dindo Classification）

Grade I: Any deviation from the ordinary postoperative course without the need for pharmacological treatment or surgical, endoscopic, and radiological interventions. Acceptable therapeutic regimens are: drugs as antiemetics, antipyretics, analgesics, diuretics, electrolytes, and physiotherapy. This grade also includes wound infections opened at the bedside.

Grade II: Requiring pharmacological treatment with drugs other than such allowed for grade I complications. Blood transfusions and total parenteral nutrition are also included.

Grade III: Requiring surgical, endoscopic or radiological intervention

IIIa: Intervention not under general anesthesia

IIIb: Intervention under general anesthesia

Grade IV: Life-threatening complication (including CNS complications)* requiring IC/ICU management

IVa: Single organ dysfunction (including dialysis)

IVb: Multiorgan dysfunction

Grade V: Death of a patient

Suffix “d”: If the patient suffers from a complication at the time of discharge, the suffix “d” (for “disability”) is added to the respective grade of complication. This label indicates the need for a follow-up to evaluate the complication fully.

* Brain hemorrhage, ischemic stroke, subarachnoidal bleeding, but excluding transient ischemic attacks.

IC: Intermediate care; ICU: Intensive care unit

(3) Blood test items (Postoperative day 1, 3, 6, 10):

- Peripheral venous blood: Hb, RBC, WBC, LYM, NEU, NEU%, PLT;
- Blood biochemical indexes: ALB, prealbumin, TBil, DBil, AST, ALT, Cr, BUN, BG, CRP;

(4) Postoperative rehabilitation evaluation items:

- First ambulation time (hour);
- First anal exsufflation/ defecation time (hour);
- Time to full/semi-liquid food intake (hour);

- Daily highest body temperature (°C);
- Chest tube extubation time (hour), daily drainage volume (ml);
- Peritoneal drainage tube extubation time (hour), daily drainage volume (ml);
- Volume of blood transfusion (defined as transfusion of red cell suspension or plasma, ml));
- Hospitalization time after operation (d).

8.6 Follow-up

8.6.1 Follow-up Period and Attentions

- Each research center should arrange a specialist to carry out the follow-up 30 days after operation (postoperative hospital stay ≤ 30 days) or operation to first discharge from hospital (postoperative hospital stay > 30 days).
- In this study, it is recommended that the follow-up examination should be conducted in the research center or a tertiary hospital, and the specialist should record the results.
- The specialist should evaluate and record the recovery situation of patient through analyzing the examination results.
- If the patient refuses the follow-up according to the protocol, it will be recorded as a case of “lost to follow-up”, and analyzed together with the cases meeting the study criteria at the end of the study (it will not be withdrawn from the PP Set).

8.6.2 Examination Items

(1) Physical Examination:

- The doctor in charge should conduct a physical examination at the time of follow-up, and be aware of the vital signs, systemic superficial lymph nodes and so on.

(2) Blood test items:

- Peripheral venous blood: Hb, RBC, WBC, LYM, NEU, NEU%, PLT；
- Blood biochemical indexes: ALB, prealbumin, TBil, DBil, AST, ALT, Cr, BUN, BG；
- Serum tumor markers: CEA, SCC；

(3) Imageological Examination:

- Thoracic enhanced CT (slice thickness of 5mm or less. If patients are allergic to the contrast agent, plain CT is permitted);
- Gastroscopy, ultrasonography, whole-body bone scan, PET-CT, etc., when attending doctors evaluate that it’s necessary.

8.7 Postoperative Adjuvant Therapy

- According to the postoperative pathological results, R0 resection cases with Stage II/III/IV should be given adjuvant chemotherapy. The chemotherapy regimen is not specified in this study.
- For relapse cases after surgical resection, the follow-up treatment protocols are

not specified in this study.

8.8 Assessments during the follow-up phase

The first follow-up visit is performed 1 month after surgery. From then on, follow-up visits are carried out every 3 months in the first two years of follow-up and every 6 months from the third year until the end of follow-up (min. 3 years). For all patients, follow-up assessment is performed until the end of the trial or death. The end of the trial will be 3 years after the study treatment of the last patient started. The detailed examination items include standard laboratory tests (blood routine, tumor biomarker), a CT scan of thorax, an ultrasound of the neck and abdomen and quality of life questionnaires (EORTC C-30+OES-18).

8.9 Definitions

8.9.1 ECOG Performance Status

Developed by the Eastern Cooperative Oncology Group:

- 0：Fully active, able to carry on all pre-disease performance without restriction.
- 1：Restricted in physically strenuous activity but ambulatory and able to carry out work of a light or sedentary nature, e.g., light housework, office work.
- 2：Ambulatory and capable of all selfcare but unable to carry out any work activities; up and about more than 50% of waking hours.

- 3：Capable of only limited selfcare; confined to bed or chair more than 50% of waking hours.
- 4：Completely disabled; cannot carry on any selfcare; totally confined to bed or chair.
- 5：Dead.

Patients at Grade 3, 4, and 5 are generally considered to be unsuitable for surgical treatment or chemotherapy.

8.9.2 ASA Classification

- ASA I: A normal healthy patient.
- ASA II: A patient with mild systemic disease.
- ASA III: A patient with severe systemic disease.
- ASA IV: A patient with severe systemic disease that is a constant threat to life.
- ASA V: A moribund patient who is not expected to survive without the operation.
- ASA VI: A declared brain-dead patient whose organs are being removed for donor purposes.

*The addition of “E” denotes Emergency surgery: (An emergency is defined as existing when delay in treatment of the patient would lead to a significant increase in the threat to life or body part)

Generally, ASA I/II patients are considered to be suitable for surgical treatment. ASA III patients are exposed to have some risks of anesthesia, and adequate preparation should be made before anesthesia. ASA IV patients are exposed to have high risks of anesthesia, and the perioperative mortality rate is very high even if the preoperative preparation is adequate. ASA V/VI patients are considered to be

unsuitable for surgical treatment.

9 Definitions of End Points

9.1 Primary outcome

Overall Survival (OS) : The primary outcome is the overall survival time in the intent-to-treat population, which ends with the date of death of any causes since the date of randomization assessed up to 36 months. For patients alive at study closure, the survival time will be censored at time of last known survival status.

9.2 Secondary outcomes

1. Progression-free survival (PFS) time: It is defined as the time from the date of randomization to the date of first recurrence/progression (local, regional or distant) or death assessed up to 36 months. Progression is examined by computed tomography (CT), positron emission tomography-computed tomography (PET-CT) and/or upper endoscopy.

2. Recurrence-free survival (RFS) time: It is defined as the time from the date of surgery to the date of first recurrence (local, regional or distant) or death assessed up to 36 months. Recurrence is examined by CT, PET-CT and/or upper endoscopy.

3. Postoperative pathologic stage:

Pathological complete response rate(pCR): Pathological complete response rate (pCR) is to be assessed in the resected specimen following neoadjuvant therapy using standardized work up of the resection specimen in the pathology department and standardized histological criteria for tumor regression grading. The degree of histomorphologic regression is clarified into four categories as follows: grade 1, no evidence of vital residual tumor cells (pathological complete response); grade 2, less than 10% vital residual tumor cells; grade 3, 10 to 50%; and grade 4, more than 50% according to previous report[23].

R0 resection rate: No vital tumor is presented at the proximal, distal, or circumferential resection margin, then it is considered to be R0 resection. If a vital tumor is shown at 1 mm or less from the proximal, distal, or circumferential resection margin, it is considered to be microscopically positive (R1).

Positive lymph nodes' number: According to pathological reports, record the number of positive lymph nodes.

Postoperative TNM stage according to the UICC TNM7 system[24].

4. Treatment related complications: Record the data according to International Consensus of Esophagectomy Complications Consensus Group (ECCG)[25]. Chemoradiation/chemotherapy-related toxicities during preoperative time are collected according to CTCAE version 4.03;

5. Postoperative mortality: 30-day postoperative mortality;

6. Quality of life(QOL): QOL is respectively evaluated at randomization, 4 weeks after neoadjuvant therapy and 1 month, 4 month, 7 month and yearly after surgery among patients by using the European Organization for Research and Treatment of Cancer Quality of Life Questionnaire C-30 (EORTC QLQ-C30) and EORTC QLQ-OES18, it is assessed up to 36 months.

10 Statistical Analyses

10.1 Definition of Analysis Set

- Intent-to-treat population (ITTP)

Cases who agreed to participate in the clinical study and signed informed consent.

- Modified intent-to-treat population (MITTP)

Cases randomly assigned to receive NCRT or nCT and with at least one record of follow-up data after surgery.

- Per-protocol population (PPP)

Cases accorded the study protocol, with good compliance, and completed CRF.

- Safety analysis population (SAP)

Cases randomly assigned to receive NCRT or nCT and with safety evaluation data after surgery.

10.2 Analysis Plan

- Statistical software: SAS statistical software.
- Descriptive statistics:
  - Continuous data: number of cases (number of missing cases), mean, median, standard deviation, P25, P75, minimum and maximum;
  - Categorical data: frequency and the corresponding percentages. For primary safety endpoint, calculate the 95% CI in addition to the percentage.
- Statistical inference: unless otherwise specified, the two-sided $P \leq 0.05$ indicates statistically significant differences between the two groups.
  - Statistical analysis for primary endpoint: the 3-year overall survival rates in the two treatment arms will be calculated by the Kaplan-Meier method and compared by the log rank test. The Cox proportional hazard model will be used to evaluate the survival-independent factors.
  - Statistical analysis for baseline variables and secondary endpoints: continuous variables were examined by independent sample t-test or Wilcoxon rank-sum test, and categorical variables were compared by Pearson chi-square test, Fisher's exact test or CMH chi-square test as appropriate.

Analysis of withdrawn patients: the number of patients who are enrolled, withdrawn, removed, completed, and number of every analysis set will be listed.

11 Data Management

11.1 Case Report Form (CRF)

11.1.1 Types and Submission Deadline

CRF used in this study and the submission deadline is as follows:

- Case screening: 7 days prior to surgery (time frame: 3 days)
- Enrolling: submitted to the data center one day prior to surgery
- Surgery: within 1 day after surgery
- Postoperation-Discharge: within 3 days after the first discharge
- Follow-up records: 7 days after each follow-up point

11.1.2 Transmission Methods

- Paper CRF and web-based eCRF form are used for data submission.

11.1.3 Amendment

After the start of the study, if the CRF is found lack of necessary data items or unclear items, under the premises of ensuring the amendment of the CRF does not cause medical and economic burden and increased risks to the selected patients, the CRF can be modified after the Research Committee adopt it through discussing at the meeting. If the amendment of the CRF does not require to modify the study protocol, this study protocol will not be modified. That whether it is necessary to submit a report or lodge an application to each research center's IRB for the CRF amendment should follow the provisions of various centers.

11.2 Monitoring and Supervision

In order to study whether the implementation follows the protocol safely, to study whether to collect the data correctly, monthly monitoring should be implemented during the period of selection of cases in principle. The monitoring is based on the hospital visit to compare the difference between and the original data and data submitted.

The periodic data report completed by the data center should be submitted to the Research Committee, the Research Responsible Person and Efficacy and Safety Evaluation Committee, and should be discussed and analyzed in accordance with relevant monitoring provisions. The regular monitoring is to aim at feedback, improving the scientific, ethical nature of the study rather than trying to expose study or hospital issues. The Research Committee, the Research Responsible Person, and the person in charge of research participating hospitals should strive to improve and to avoid the problems pointed out in the regular monitoring reports.

11.2.1 Monitoring Items

- Data collection completed status: Selected registration number (cumulative/different time of period, all hospitals/different hospitals)
- Eligibility: Ineligible patients/potentially ineligible patients (different hospitals)
- Different end of treatment, the reasons for suspension/end (different hospitals) in the study protocol
- Background factors, pre-treatment report factors, post-treatment report factors when selected for registration
- Severe adverse events (different hospitals)
- Adverse events/adverse reactions (different hospitals)
- Proportion of conversion to open surgery (different hospitals)
- Protocol deviation (different hospitals)

- Progress and safety of the study, other issues

11.2.2 Acceptable Range of Adverse Events

Based on the qualification of the research centers in this study, in general, treatment-related death and life-threatening complications caused by surgeries do not happen basically; the percent of more than 3% is considered unacceptable. If treatment-related death is suspected or non-hematologic Grade 4 toxicity having a causal relationship with the surgery is determined, adverse events on each patient should be respectively reported to the Efficacy and Safety Evaluation Committee. If the number of treatment-related deaths or the number of patients with determined non-hematologic Grade 4 toxicity having a causal relationship with the surgery is up to 4, the final incidence proportion of adverse events will be apparently more than 3%, and therefore the inclusion of patients must be immediately suspended. Whether the study can continue to proceed should be determined until reviewed by the PI Efficacy and Safety Evaluation Committee.

11.2.3 Deviation/Violation of Study Protocol

Surgical resection, clinical examinations, or toxicity, efficacy evaluation and so on failing to be conducted in accordance with the study protocol are the deviation of the study protocol. When the monitoring is carried out, deviations developed by the Data Center and Research Committee in advance (allowed to after the start of the study in special circumstances) beyond the acceptable range specified in each study center should be included in the monitoring report in the form of “cases of deviation possibility”, and divided into any arbitrary one of the following after discussed by the Research Committee:

11.2.3.1 Violation

Clinically inappropriate, a deviation at least complying with one of the following items specified in the protocol is called “violation”

(1) Affecting the study endpoint evaluation

(2) The responsibility lays the doctor in charge/hospital

(3) Intentional or systematic

(4) Significant danger or the degree of deviation

Papers should record content violation in principle.

11.2.3.2 Acceptable deviation

- The acceptable deviation represents the acceptable range of each item set by the Research Representative/Committee and the data center before or after the beginning of the study.
- If it is within an acceptable range of deviation set in advance, no record is required in the monitoring report.

11.2.3.3 Deviation

- Items that do not comply with 11.2.3.1 or with 11.2.3.2 are deviation items.
- Specific deviations that occur several times should be recorded as much as possible when the paper is published.
- When the monitoring report is discussed, the deviation should be classified as the following:

  (1) Deviated from undesired results: should be reduced

  (2) Deviation (inevitable): not to be actively reduced

(3) Deviation (clinically appropriate): positive affirmation of the judgment by the doctor in charge/ hospital

12 Provisions on Adverse Events

The evaluation in this study refers to CTCAE v4.0 and "Accordion Severity Grading System"

12.1 Evaluation

- Evaluation of adverse event/adverse reaction comprehensively refers to the [Accordion Severity Grading System] and [CTCAE v4.0].
- Adverse events are graded according to the content that is the nearest Grade 0 ~ 4 definition. For treatment-related death, death adverse events are classified as Grade 5 in the original CTCAE.
- Toxicity items specified in the, Grade, and the discovery date of Grade should be recorded in the treatment process report. For other toxicity items observed, observed Grade 3 toxicity items are only recorded the freedom registration column of the treatment process report, as well as Grade and the discovery date of Grade. The grade recorded in the treatment process report must be recorded in the case.
- CTCAE v4.0, the so-called "Adverse Event", "all observed, unexpected bad signs, symptoms and diseases(abnormal value of clinical examination are also included) in the treatment or disposal, regardless of a causal relationship with the treatment or intervention. So it can be divided into two types based on whether there is a causal relationship or not.
- Therefore, even if events that "obviously caused by primary disease (cancer)" or caused by

supportive therapy or combination therapy rather than the study regimen treatment (protocol treatment) are defined as “adverse events”.

- For adverse event data collection strategy, the following principle should be complied with in this study: Adverse events within 30 days from the last treatment day of the study regimen or hospitalization before first discharge (postoperative hospital stay > 30 days) (protocol treatment) should be collected entirely, regardless of the presence or absence of a causal relationship. (When adverse events are reported, the causality and classification of adverse events are separately discussed)

12.2 Reporting

- When “severe adverse events” or “unexpected adverse events” occur, the Research Responsible Person of each research center should report to the Research Committee/PI (Lijie Tan). Before the start of the study, the Research Committee should send the report template to each research center in advance. When “severe adverse events” or “unexpected adverse events” occur, the Research Responsible Person of each research center should report them to the Research Committee/PI (Lijie Tan).
- Adverse events based on the relevant laws and regulations should be reported to the province (city) Health Authority at the location of each research center. Severe adverse events based on clinical research-related ethical guidelines should be reported to the person in overall charge of the medical institution. The appropriate reporting procedures should be completed in accordance with the relevant provisions of all medical institutions at the same time. The person in charge of each center should hold obligations and responsibility for the emergency treatment of patients with any degree of adverse events to ensure patient safety.

12.2.1 Adverse Events with Reporting Obligations

12.2.1.1 Adverse Events with Emergency Reporting Obligations

Any of the following adverse events is the object that any adverse event should be reported urgently to:

- All patients died during the course of treatment or within 30 days from the last treatment day, regardless of the presence or absence of a causal relationship with the study regimen treatment. If cases are withdrawn of treatment, even if the latter treatment has begun, those patients also belong to emergent reporting objects, as long as within 30 days from the last treatment day or during hospitalization (hospital stay > 30 days). (day 0 is the final treatment day and 30 days is starting from the next day)
- Those patients with unexpected Grade 4 non-hematologic toxicity (CTCAE v4.0 adverse events other than the blood/bone marrow group), having a causality with the treatment (any of definite, probable, possible) are also emergent reporting objects.

12.2.1.2 Adverse Events with Regular Reporting Obligations

Any one of the following adverse events is a regular reporting object:

(1) After 31 days from the last treatment day, death that cannot rule out the causal relationship with treatments, including suspected treatment-related death; death due to apparent primary disease is excluded.

(2) Expected Grade 4 non-hematologic toxicity (CTCAE v4.0 adverse events other than the blood/bone marrow group).

(3) Unexpected Grade 3 adverse events: Grade 3 adverse events are not recorded in the **12.1 expected adverse events**.

(4) Other significant medical events: adverse events that the study group deems are found to bring essential and potentially permanent, significant impact on their offspring (except for MDS

myelodysplastic syndrome, and secondary cancer)

Adverse events among above (2)-(4), determined to have a causal relationship (any of definite, probable, and possible) with the study regime are regular reporting objects.

12.2.2 Reporting Procedure

12.2.2.1 Emergency Reporting

- When emergent adverse events of emergency study reporting objects happened, the doctor in charge will quickly report it to the Research Responsible Person of the research participating hospitals. Where no contact can be gotten with the Research Responsible Person of the hospital, the coordinator, or the doctor in charge of the hospital must perform the responsibility instead.
- First Reporting: Within 72 hours after the occurrence of adverse events, the Research Responsible Person of the hospital should complete the “AE/AR/ADR first emergency report” and send it to the Research Committee by FAX and telephone.
- Second Reporting: The Research Responsible Person of each research participating hospital completes the “AE/AR/ADR Report” and a more detailed case information report (A4 format), and then fax the two reports to the Research Committee within 15 days after the occurrence of adverse events. If any autopsy examination, the autopsy result report should be submitted to the Research Committee.

12.2.2.2 General Reports

- The Research Responsible Person of each research participating hospital completes the “AE/AR/ADR report”, and then fax it to the Research Committee within 15 days after the occurrence of adverse events.

12.3 Responsibilities and Obligations

12.3.1 Judgment of Study Discontinuation and Necessity for Sending an Emergency Notice to the Hospital

After the receipt of the report of the Research Responsible Person of the research participating hospital, the Research Committee reply to the Research Responsible Person of the unit for confirmation and negotiation, and then they jointly determine the urgency, importance, and influence of reporting events; if necessary, they temporarily stop the study, and contact with all research participating hospitals to take emergency notification countermeasures. According to the severity of urgency, data center and research participating hospitals can be contacted by telephone or instrument FAX as soon as possible after the initial contact by phone.

12.3.2 Report to PI Efficacy and Safety Evaluation Committee

After notifying, discussing and clarifying the adverse events in line with **12.2.1 adverse events with reporting obligations** in the emergency reports or regular reports to the Research Responsible Person of research participating units, the Research Committee should submit a report to the Efficacy and Safety Evaluation Committee within 3 days after the occurrence of adverse events and request a review that whether the reason analysis of and solution to the adverse events by the Research Responsible Person are appropriate..

At that time, “AE/AR/ADR First Emergency Report” and “AE/AR/ADR Report” submitted by the research participating hospital should include the discussion results and countermeasures of the Research Committee/Research Responsible Person(including the judgment of research continue/discontinue). For death within 30 days, treatment-related death among death after 31 days and expected Grade 4 non-hematologic toxicity, not only the course of individual patient are included, but also consideration given to that whether the frequency of occurrence falls within the

expected range are included. If the frequency of occurrence exceeds the expected range, it should be faithfully recorded in the “II classification of adverse events-others” of “AE/AR/ADR Report”.

12.3.3 Notice to the Research Participating Hospitals

After submitting the report to the CLASS Efficacy and Safety Evaluation Committee, the Research Committee/Research Responsible Person should notify the efficacy, and review, proposal content of the Efficacy and Safety Evaluation Committee in written form to all research participating hospitals.

If failing to submit the report to the Efficacy and Safety Evaluation Committee, the Research Committee/Research Responsible Person should report their judgment in written form to the Research Responsible Person of a research participating hospital that submitted the report.

12.3.4 Discussion of Adverse Events under Regularly Monitoring

During the regular monitoring, the Research Committee/Research Responsible Person should carefully discuss, study adverse events in the monitoring report submitted by the research data center to confirm no missing report by each research participating hospital. The existence or inexistence of under-reporting adverse events should be clearly documented in the discussion results of [regularly monitoring report] of the Research Committee.

12.4 Review of Efficacy and Safety Evaluation Committee

The Efficacy and Safety Evaluation Committee reviews and discusses the report in accordance with the procedures recorded in the Clinical Safety Information Management Guideline, and raises the recommendations in written form for the Research Responsible Person, including whether to continue to enroll the study objects or whether to need to modify the study

protocol.

13 Ethics

13.1 Responsibilities of Investigators

The investigators are responsible for the implementation of this study in its center. The investigators will ensure the implementation of this study in accordance with the study protocol and in compliance with the Declaration of Helsinki, as well as domestic and international ethical guiding principles and applicable regulatory requirements. It is especially noted that the investigators must ensure that subjects giving the written informed consent can be enrolled in this study only.

13.2 Information and Informed Consent of Subjects

An unconditional prerequisite for subjects to participate in this study is his/her written informed consent. The written informed consent of subjects participating in this study must be given before study-related activities are conducted.

Therefore, before obtaining informed consent, the investigators must provide sufficient information to the subjects. In order to obtain informed consent, the investigators will provide the information page of subjects, and the information required to comply with the applicable regulatory requirements. While providing written information, the investigators will orally inform the subjects of all the relevant circumstances of this study. In this process, the words used must be fully, easily understood by non-professionals, so that they can sign on the informed consent form according to their willingness based on subjects' fully understanding of this study.

The informed consent form must be signed and dated personally by the subjects and investigators. All subjects will be asked to sign on the informed consent form to prove that they

agree to participate in the study. The signed informed consent form with signature and date should be kept in the research center where the investigators are located and must be properly safe kept for the future review at any time during the audit, inspection, inspection period. Before participating in the study, the subjects should provide a copy of signed and dated informed consent form.

At any time, as long as access to important new information that may be related to the consent of the subjects, the investigators will revise the information pages and any other written information provided to the subjects and re-submit them to the IEC/IRB for review and raising a favorable opinion. The revised information agreed will be provided to each subject participating in the study. The researchers will explain the changes made to the previous version of ICF to the subjects.

13.3 Identity and Privacy of Subjects

After obtaining an informed consent form, each selected subject is assigned with subject number (Allocation Number, AN). This number will represent the identity of the subject in the whole study and the clinical research database for the study. The collected data of subjects in the study will be stored in the ID.

In the entire study, various safety measures to minimize leaking risks in the utilization process of personal information will be taken, including: (1) only the investigators were able to link the research data of the subjects with themselves through the identify table kept in the research center after authorized; (2) in the raw data auditing on-site conducted by the supervisors of this study, as well as relevant inspection and inspection visit by the supervision departments, the personnel engaging above activities may view the original medical information of subjects that will be kept strictly confidential.

Data collection, transmission, handling, and storage of subjects will comply with the data protection and privacy regulations. This corresponding information will be provided to the subjects, and the subjects were asked to provide their consent for the treatment procedures of

above data in accordance with national regulations.

13.4 Independent Ethics Committee or Institutional Review Committee

Before beginning the study, the Research Center will be responsible for submitting the study protocol and relevant documents (informed consent form, subject information page, CRF, and other documents that may be required) to the Independent Ethics Committee (IEC)/ Institutional Review Board (IRB) to obtain their favorable opinion/approval. The favorable opinion/approval documents of the IEC/IRB will be archived in the research center folders of the investigators.

Before obtaining the written proof of favorable opinions/approval of the IEC/IRB, the investigators are forbidden to begin the study in the center. The IEC/IRB will be asked to provide the written proof of the date of the favorable opinions/approval meeting and the written proof of the members presenting at the meeting and voting members. The IEC/IRB should provide the written proof of the favorable opinion/approval, recording the reviewed study, protocol version, and Informed Consent Form version. If possible, a copy of the minutes should also be obtained.

In the case of major revisions in this study, the amendment of the study protocol will be submitted to the IEC/ IRB prior to performing. In the course of the study, the relevant safety information will be submitted to the IEC/IRB in accordance with national regulations and requirements.

13.5 Supervisory Authority

The study protocol and any relevant documents (for example, the study protocol, the subject's informed consent form) will be submitted according to the Ethical Review Approach of Biomedical Research Involving Human Beings (Trial) (2007) and the applicable regulatory requirements of our country or will notify the ethical review guidance counseling organization of the provincial health administrative departments at the location of each research center.

14 Organizations and Responsibilities of Study

14.1 Research Committee

- Being responsible for developing study protocol, auditing eligibility for inclusion, and guiding the interpretation of informed consent; being responsible for the collection of hazardous/adverse event reports, guiding the clinical diagnosis and treatment of such events, and the emergency intervention of serious adverse events.
- The PI of Research Committee: Lijie Tan (Department of Thoracic Surgery, Zhongshan Hospital, Fudan University). Add: Department of Thoracic Surgery, Zhongshan Hospital, Fudan University, Fenglin Road 180, Shanghai 200032, China; Tel: 86-21-64041990-2917; Fax: 86-21-64038477; Mobile: 13681972151; E-mail: lijie.tan@zs-hospital.sh.cn.
- Research Representative: Hao Wang (Department of Thoracic Surgery, Zhongshan Hospital, Fudan University). Add: Department of Thoracic Surgery, Zhongshan Hospital, Fudan University, Fenglin Road 180, Shanghai 200032, China; Tel: 86-21-64041990-2917; Fax: 86-21-64038477; Mobile: 13816051785; E-mail: wang.hao@zs-hospital.sh.cn.

- Research centers to participate in this study:

PI	Title	Research Center
Lijie Tan	Professor	Zhongshan Hospital, Fudan University
**CI**	**Title**	**Research Center**
Ming Du	Professor	the First Affiliated Hospital of Chongqing Medical University, Chongqing
Hongjing Jiang	Professor	Tianjin Medical University Cancer Institute and Hospital, Tianjin
Zhigang Li	Professor	Shanghai Chest Hospital, Shanghai Jiao Tong University, Shanghai,
Deyao Xie	Professor	the First Affiliated Hospital of Wenzhou Medical University
Changhong Lian	Professor	Heping Hospital Affiliated to Changzhi Medical College, Changzhi, Shanxi
Deyao Xie	Professor	Fujian Medical University Union Hospital, Fuzhou, Fujian
Deyao Xie	Professor	Union Hospital, Tongji Medical College, Huazhong University of Science and Technology, Wuhan, Hubei
Hecheng Li	Professor	Ruijin Hospital, Shanghai Jiao Tong University School of Medicine, Shanghai
Ke-Neng Chen	Professor	the First Department of Thoracic Surgery, Peking University Cancer Hospital and Institute, Peking University School of Oncology

14.2 Efficacy and Safety Evaluation Committee

- Being responsible for the supervision, monitoring of the treatment safety, and therapeutic

efficacy of this study.

- The PI of Efficacy and Safety Evaluation Committee: Lijie Tan (Department of Thoracic Surgery, Zhongshan Hospital, Fudan University)

14.3 Data Center

- Participating in the design of this study protocol, being responsible for data analysis, statistical interpretation, and issuing of statistical reports.
- Being responsible for the formulation and provision of CRFs and eCRF (web-based electronic case report forms) and management, storage of research data, and maintenance of database.
- Person in charge of Data Center: Professor Zhao Naiqing (Department of Biological Statistics, Fudan University)
- The Second Person in Charge of Management of Study Data: CRO

14.4 Data and Safety Monitoring Board

- DSMB is responsible for the supervision of efficacy, the safety of this study, supervising of all aspects performed of the study, and licensing before the release of the validity of the study results.
- Person in Charge of DSMB: CRO

14.5 Independent Ethics Committee/Institutional Review Board (IEC/IRB)

- Being Responsible for evaluating this study in order to determine "whether to minimize risks that the subjects are exposed to" and "whether the risks that the subjects are exposed to are reasonable compared to expected benefits".
- The independent Ethics Committee/Institutional Review Board (IEC/IRB) at the location of each research center is responsible for the ethics review of all research participating units.

15 Publications of Research Results

- The publication of the research results of the paper should follow the established principle of the publication period in the study protocol.
- When there are no definite established policies of the research group, the publication of the paper should follow the following principle: the main statistical analysis, the final statistical analysis, and the final complete public paper written contributions to journals in English. Unless clearly provided in the study protocol, the methods of used statistical analysis and the final statistical analysis cannot be published without approval of the Efficacy and Safety Evaluation Committee. However, excluding the results of the final statistical analysis of this study, the research representative or the Research Committee can publish the Society Paper (Abstract) to introduce of this study just need to obtain consent from the person in charge of the data center.
- In principle, the author of the main published paper of the research results is firstly the Research Committee, followed by the research representative, the person in charge of statistics of the data center (the person in charge of statistical analysis for publication). The rest should follow the paper written contribution rules. In order of the selected registration size of samples, the Research Responsible Persons of all research centers are listed as co-authors. All co-authors shall review the paper and agree to publish it before the paper

submission. If the consent cannot be gotten from an investigator because of disagreeing with the published content, the research representative has the right not to list the investigator as co-author.

- For the overall data collected in this study, if any person in charge of research center need make a secondary analysis or make an analysis for other research purposes, the consent of the Research Committee shall be gotten; when a person in charge of research center need to use the data of his group to make the speech on the academic conference, the data source should be noted and informed the Research Committee.
- The publication of the primary objectives should be penned by people in charge of the research, principally. The publication of the second objectives or secondary analysis for the results can be negotiated by the person in charge of research participating units of this research organization but must obtain the permission of the person in charge of the whole research.
- The person in charge of the research center has right to save their single-center data but should follow the privacy principles; For the results, form, the content of published single-center data, the relevant responsibilities should be at their own risk. The Research Committee does not assume any responsibility; the use of single-center data must be informed and obtain the recognized accuracy from the CLASS data center; the single-center data of statistical analysis must be marked to derive from this study of the CLASS in order to avoid repeat inclusion at the time of systemic analysis.
- Without the approval of both the Research Committee and the data center, No Research Committee personnel cannot directly obtain the overall data and results of statistical analysis of this study from the data center.

16 References

1. Jemal A, Bray F, Center MM, Ferlay J, Ward E, Forman D: Global cancer statistics. CA: a cancer journal for clinicians 2011, 61(2):69-90.

2. Chen W, Zheng R, Baade PD, Zhang S, Zeng H, Bray F et al: Cancer statistics in China, 2015. CA: a cancer journal for clinicians 2016, 66(2):115-132.

3. van Hagen P, Hulshof MC, van Lanschot JJ, Steyerberg EW, van Berge Henegouwen MI, Wijnhoven BP et al: Preoperative chemoradiotherapy for esophageal or junctional cancer. The New England journal of medicine 2012, 366(22):2074-2084.

4. Gebski V, Burmeister B, Smithers BM, Foo K, Zalcberg J, Simes J et al: Survival benefits from neoadjuvant chemoradiotherapy or chemotherapy in oesophageal carcinoma: a meta-analysis. The Lancet Oncology 2007, 8(3):226-234.

5. Jang R, Darling G, Wong RK: Multimodality approaches for the curative treatment of esophageal cancer. Journal of the National Comprehensive Cancer Network : JNCCN 2015, 13(2):229-238.

6. Sjoquist KM, Burmeister BH, Smithers BM, Zalcberg JR, Simes RJ, Barbour A et al: Survival after neoadjuvant chemotherapy or chemoradiotherapy for resectable oesophageal carcinoma: an updated meta-analysis. The Lancet Oncology 2011, 12(7):681-692.

7. Tepper J, Krasna MJ, Niedzwiecki D, Hollis D, Reed CE, Goldberg R et al: Phase III trial of trimodality therapy with cisplatin, fluorouracil, radiotherapy, and surgery compared with surgery alone for esophageal cancer: CALGB 9781. Journal of clinical oncology : official journal of the American Society of Clinical Oncology 2008, 26(7):1086-1092.

8. Mariette C, Dahan L, Mornex F, Maillard E, Thomas PA, Meunier B et al: Surgery alone versus chemoradiotherapy followed by surgery for stage I and II esophageal cancer: final analysis of randomized controlled phase III trial FFCD 9901. Journal of clinical oncology : official journal of the American Society of Clinical Oncology 2014, 32(23):2416-2422.

9. Kumagai K, Rouvelas I, Tsai JA, Mariosa D, Klevebro F, Lindblad M et al: Meta-analysis of postoperative morbidity and perioperative mortality in patients receiving neoadjuvant chemotherapy or chemoradiotherapy for resectable oesophageal and gastro-oesophageal junctional cancers. The British journal of surgery 2014, 101(4):321-338.

10. Ando N, Kato H, Igaki H, Shinoda M, Ozawa S, Shimizu H et al: A randomized trial comparing postoperative adjuvant chemotherapy with cisplatin and 5-fluorouracil versus preoperative chemotherapy for localized advanced squamous cell carcinoma of the thoracic esophagus (JCOG9907). Annals of surgical oncology 2012, 19(1):68-74.

11. Baba Y, Watanabe M, Yoshida N, Baba H: Neoadjuvant treatment for esophageal squamous cell carcinoma. World journal of gastrointestinal oncology 2014, 6(5):121-128.

12. Biere SS, van Berge Henegouwen MI, Maas KW, Bonavina L, Rosman C, Garcia JR et al: Minimally invasive versus open oesophagectomy for patients with oesophageal cancer: a multicentre, open-label, randomised controlled trial. Lancet 2012, 379(9829):1887-1892.

13. Luketich JD, Alvelo-Rivera M, Buenaventura PO, Christie NA, McCaughan JS, Litle VR et al: Minimally invasive esophagectomy: outcomes in 222 patients. Annals of surgery 2003, 238(4):486-494; discussion 494-485.

14. Luketich JD, Pennathur A, Awais O, Levy RM, Keeley S, Shende M et al:

Outcomes after minimally invasive esophagectomy: review of over 1000 patients. Annals of surgery 2012, 256(1):95-103.

15. Bakhos C, Oyasiji T, Elmadhun N, Kent M, Gangadharan S, Critchlow J et al: Feasibility of minimally invasive esophagectomy after neoadjuvant chemoradiation. Journal of laparoendoscopic & advanced surgical techniques Part A 2014, 24(10):688-692.

16. Warner S, Chang YH, Paripati H, Ross H, Ashman J, Harold K et al: Outcomes of minimally invasive esophagectomy in esophageal cancer after neoadjuvant chemoradiotherapy. The Annals of thoracic surgery 2014, 97(2):439-445.

17. Burmeister BH, Thomas JM, Burmeister EA, Walpole ET, Harvey JA, Thomson DB et al: Is concurrent radiation therapy required in patients receiving preoperative chemotherapy for adenocarcinoma of the oesophagus? A randomised phase II trial. European journal of cancer 2011, 47(3):354-360.

18. Stahl M, Walz MK, Stuschke M, Lehmann N, Meyer HJ, Riera-Knorrenschild J et al: Phase III comparison of preoperative chemotherapy compared with chemoradiotherapy in patients with locally advanced adenocarcinoma of the esophagogastric junction. Journal of clinical oncology : official journal of the American Society of Clinical Oncology 2009, 27(6):851-856.

19. Feng M, Shen Y, Wang H, Tan L, Zhang Y, Khan MA et al: Thoracolaparoscopic esophagectomy: is the prone position a safe alternative to the decubitus position? Journal of the American College of Surgeons 2012, 214(5):838-844.

20. Shen Y, Feng M, Khan MA, Wang H, Tan L, Wang Q: A simple method minimizes chylothorax after minimally invasive esophagectomy. Journal of the American College of Surgeons 2014, 218(1):108-112.

21. Shen Y, Zhang Y, Tan L, Feng M, Wang H, Khan MA et al: Extensive mediastinal lymphadenectomy during minimally invasive esophagectomy: optimal results from a single center. Journal of gastrointestinal surgery : official journal of the Society for Surgery of the Alimentary Tract 2012, 16(4):715-721.

22. Shimoda T: [Japanese classification of esophageal cancer, the 10th edition--Pathological part]. Nihon rinsho Japanese journal of clinical medicine 2011, 69 Suppl 6:109-120.

23. Becker K, Mueller JD, Schulmacher C, Ott K, Fink U, Busch R et al: Histomorphology and grading of regression in gastric carcinoma treated with neoadjuvant chemotherapy. Cancer 2003, 98(7):1521-1530.

24. Edge SB, Compton CC: The American Joint Committee on Cancer: the 7th edition of the AJCC cancer staging manual and the future of TNM. Annals of surgical oncology 2010, 17(6):1471-1474.

25. Low DE, Alderson D, Cecconello I, Chang AC, Darling GE, D'Journo XB et al: International Consensus on Standardization of Data Collection for Complications Associated With Esophagectomy: Esophagectomy Complications Consensus Group (ECCG). Annals of surgery 2015, 262(2):286-294.

26. Shapiro J, van Lanschot JJ, Hulshof MC, van Hagen P, van Berge Henegouwen MI, Wijnhoven BP et al: Neoadjuvant chemoradiotherapy plus surgery versus surgery alone for oesophageal or junctional cancer (CROSS): long-term results of a randomised controlled trial. The Lancet Oncology 2015, 16(9):1090-1098.
