# Supplementary material for: Morbidity and Mortality of Patients Who Underwent Minimally Invasive Esophagectomy After Neoadjuvant Chemoradiotherapy vs Neoadjuvant Chemotherapy for Locally Advanced Esophageal Squamous Cell Carcinoma: A Randomized Clinical Trial
Source: JAMA Surg. 2021 Mar 17;156(5):444–51. doi: 10.1001/jamasurg.2021.0133 (PMC7970392; doi:10.1001/jamasurg.2021.0133)
Supplement: Supplement 2. — eTable 1. Eligibility Criteria for Enrolling Patients eTable 2. The Baseline Clinical Characteristics of Patients eTable 3. Adverse Events During Neoadjuvant Therapy eFigure. The Overall Survival Curve of the Two Groups (Kaplan–Meier Plots) [file jamasurg-e210133-s002.pdf]

Supplementary Online Content

Wang H, Tang H, Fang Y, et al. Morbidity and mortality of patients who underwent minimally invasive esophagectomy after neoadjuvant chemoradiotherapy vs neoadjuvant chemotherapy for locally advanced esophageal squamous cell carcinoma: a randomized clinical trial. *JAMA Surg.* Published online March 17, 2021. doi:10.1001/jamasurg.2021.0133

eTable 1. Eligibility Criteria for Enrolling Patients
eTable 2. The Baseline Clinical Characteristics of Patients
eTable 3. Adverse Events During Neoadjuvant Therapy
eFigure. The Overall Survival Curve of the Two Groups (Kaplan–Meier Plots)

This supplementary material has been provided by the authors to give readers additional information about their work.

© 2021 Wang H et al. *JAMA Surgery*.

eTable 1. Eligibility Criteria for Enrolling Patients

**Inclusion**
1. Histologically-confirmed squamous cell carcinoma of the esophagus;
2. Tumors of the esophagus are located in the thoracic cavity;
3. Pre-treatment stage as cT3-4aN0-1M0 (AJCC/UICC 7th Edition) (In case of stage cT4a, curative resectability has to be explicitly verified by the local surgical investigator prior to randomization).
4. Age is between 18 years and 75 years,
5. Eastern Cooperative Oncology Group (ECOG) performance status 0–1;
6. Adequate cardiac function. All patients should perform ECG, and those with a cardiac history or ECG abnormality should perform echocardiography with the left ventricular ejection fraction >50%.
7. Adequate respiratory function with FEV1 ≥ 1.2 L, FEV1% ≥ 50% and DLCO ≥ 50% shown in pulmonary function tests.
8. Adequate bone marrow function (White Blood Cells >4 × 10^9 /L; Neutrophil >2.0 × 10^9 /L; Hemoglobin >90 g/L; platelets > 100 × 10^9 /L);
9. Adequate liver function (Total bilirubin <1.5× Upper Level of Normal (ULN); Aspartate transaminase(AST) and Alanine transaminase (ALT) <1.5× ULN);
10.Adequate renal function (Glomerular filtration rate (CCr) >60 ml/min; serum creatinine (SCr) ≤120 μmol/L);
11.The patient has provided written informed consent and is able to understand and comply with the study;
**Exclusion**
1. Patients with non-squamous cell carcinoma histology;
2. Patients with advanced inoperable or metastatic esophageal cancer;
3. Pre-treatment stage as cT1-2 N0-1 M0 (AJCC/UICC 7th Edition);
4. Pre-treatment stage as cN2–3 or cT4b (non-curativelyresectable verified by the local surgical investigator, AJCC/UICC 7th Edition);
5. Patients with another previous or current malignant disease which is likely to interfere with treatment or the assessment of response in the judgement of the local surgical investigator.
6. Any patient with a significant medical condition which is thought unlikely to tolerate the therapies. Such as cardiac disease (e.g. symptomatic coronary artery disease or myocardial infarction within last 12 months), clinically-significant lung disease, clinically-significant bone marrow, liver, renal function disorder;
7. Pregnant or lactating women and fertile women who will not be using contraception during the trial;
8. Allergy to any drugs;
9. Participation in another intervention clinical trial with interference to the chemotherapeutic or chemoradiotherapeutic intervention during this study or during the last 30 days prior to informed consent;
10.Expected lack of compliance with the protocol.

Abbreviations: AJCC, American Joint Committee on Cancer; ECOG PS, Eastern Cooperative Oncology Group performance status; FEV1, forced expiratory volume in 1 second.

© 2021 Wang H et al. *JAMA Surgery*.

eTable 2. The Baseline Clinical Characteristics of Patients

	Mean (SD)		*P* value
	nCRT group (n=132)	nCT group (n=132)	
Sex, No. (%)			0.293
Male	116 (87.9)	110 (83.3)	
Female	16 (12.1)	22 (16.7)	
Age, years	61.2 (6.7)	61.5 (6.8)	0.746
ECOG PS, No. (%)			0.746
0	110 (83.3)	108 (81.8)	
1	22 (16.7)	24 (18.2)	
Comorbidity, No. (%)			0.505
None	89 (67.4)	94 (71.2)	
One or more	43 (32.6)	38 (28.8)	
Tumor location, No. (%)			0.475
upper	16 (12.1)	13 (9.8)	
middle	89 (67.4)	84 (63.6)	
lower	27 (20.5)	35 (26.5)	
Clinical stage			0.720
cT3N0M0	27 (20.5)	22 (16.7)	
cT3N1M0	68 (51.5)	69 (52.3)	
cT4aN0M0	10 (7.6)	8 (6.1)	
cT4aN1M0	27 (20.5)	33 (25.0)	

NOTE: Tumor stage according to the American Joint Committee on Cancer, 8th Edition.

Abbreviations: BMI, body mass index; ECOG PS, Eastern Cooperative Oncology performance status; SD, standard deviation.

© 2021 Wang H et al. *JAMA Surgery*.

eTable 3. Adverse Events During Neoadjuvant Therapy

	nCRT group (n=131*)	nCT group (n=130*)	*P* value
Grade 1 and 2, No. (%)			
Anemia	52 (39.7)	49 (37.7)	0.740
Granulocytopenia	89 (67.9)	82 (63.1)	0.409
Thrombocytopenia	43 (32.8)	41 (31.5)	0.824
Anorexia	79 (60.3)	81 (62.3)	0.740
Vomiting	91 (69.5)	95 (73.1)	0.519
Pulmonary infection	6 (4.6)	3 (2.3)	0.505
Radiation esophagitis	6 (4.6)	0	0.040
Liver dysfunction	27 (20.6)	25 (19.2)	0.780
Diarrhea	6 (4.6)	8 (6.2)	0.573
Constipation	12 (9.2)	13 (10.0)	0.818
Fatigue	17 (13.0)	20 (15.4)	0.577
Fever without infection	6 (4.6)	4 (3.1)	0.756
Grade 3 and 4, No. (%)			
Granulocytopenia	10 (7.6)	7 (5.4)	0.452
Anorexia	1 (0.8)	1 (0.8)	1.000
Vomiting	1 (0.8)	1 (0.8)	1.000
Pulmonary infection	3 (2.3)	0	0.246
Esophageal perforation	4 (3.1)	0	0.131
Liver dysfunction	1 (0.8)	0	1.000
Total	20 (15.3)	9 (6.9)	0.030

NOTE. Data are presented as No. (%). Adverse events were graded according to the National Cancer Institute's Common Terminology Criteria for Adverse Events, version 5.0. Severe Adverse Events

*One of 132 patients in Group nCRT and 2 of 132 patients in Group nCT declined to receive treatment.

© 2021 Wang H et al. *JAMA Surgery*.

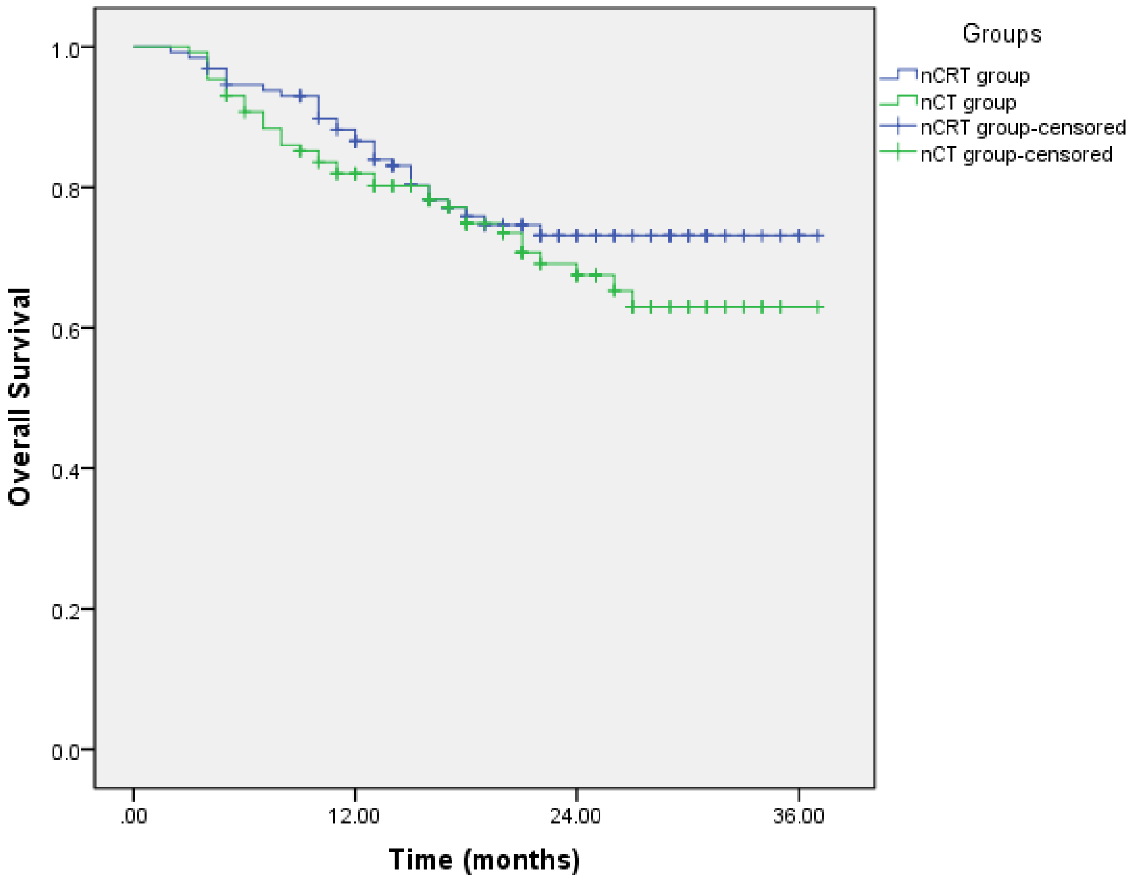

eFigure. The Overall Survival Curve of the Two Groups (Kaplan–Meier Plots)

© 2021 Wang H et al. *JAMA Surgery*.
