# Supplementary material for: Morbidity and Mortality of Patients Who Underwent Minimally Invasive Esophagectomy After Neoadjuvant Chemoradiotherapy vs Neoadjuvant Chemotherapy for Locally Advanced Esophageal Squamous Cell Carcinoma: A Randomized Clinical Trial
Source: JAMA Surg. 2021 Mar 17;156(5):444–51. doi: 10.1001/jamasurg.2021.0133 (PMC7970392; doi:10.1001/jamasurg.2021.0133)
Supplement: Supplement 3. — Data Sharing Statement [file jamasurg-e210133-s003.pdf]

Data Sharing Statement

Wang. Morbidity and Mortality of Patients Who Underwent Minimally Invasive Esophagectomy After Neoadjuvant Chemoradiotherapy vs Neoadjuvant Chemotherapy for Locally Advanced Esophageal Squamous Cell Carcinoma. *JAMA Surg*. Published March 17, 2021. doi:10.1001/jamasurg.2021.0133

Data
Data available: Yes
Data types: Deidentified participant data, Participant data with identifiers, Data dictionary
How to access data: tan.lijie@zs-hospital.sh.cn (Corresponding author)
When available: With publication

Supporting Documents
Document types: Statistical/analytic code, Informed consent form
How to access documents: tan.lijie@zs-hospital.sh.cn (Corresponding author)
When available: With publication

Additional Information
Who can access the data: Lijie Tan (Corresponding author)
Types of analyses: by email
Mechanisms of data availability: with investigator support
